# PDGF-mediated mesenchymal transformation renders endothelial resistance to anti-VEGF treatment in glioblastoma

**DOI:** 10.1038/s41467-018-05982-z

**Published:** 2018-08-27

**Authors:** Tianrun Liu, Wenjuan Ma, Haineng Xu, Menggui Huang, Duo Zhang, Zhenqiang He, Lin Zhang, Steven Brem, Donald M. O’Rourke, Yanqing Gong, Yonggao Mou, Zhenfeng Zhang, Yi Fan

**Affiliations:** 1 Department of Radiation Oncology, University of Pennsylvania Perelman School of Medicine, Philadelphia, PA 19104 USA; 2 Division of Head and Neck Surgery, Department of Otorhinolaryngology, The Sixth Affiliated Hospital of Sun Yat-sen University, Guangzhou, 510655 China; 3 Department of Medical Oncology, State Key Laboratory of Oncology in South China & Collaborative Innovation Center for Cancer Medicine, Sun Yat-sen University Cancer Center, Guangzhou, 510060 China; 4 Department of Neurosurgery, State Key Laboratory of Oncology in South China & Collaborative Innovation Center for Cancer Medicine, Sun Yat-sen University Cancer Center, Guangzhou, 510060 China; 5 Department of Obstetrics & Gynecology, University of Pennsylvania Perelman School of Medicine, Philadelphia, PA 19104 USA; 6 Department of Neurosurgery, University of Pennsylvania Perelman School of Medicine, Philadelphia, PA 19104 USA; 7 Division of Human Genetics and Translational Medicine, Department of Medicine, University of Pennsylvania Perelman School of Medicine, Philadelphia, PA 19104 USA; 8 Department of Radiology, The Second Affiliated Hospital of Guangzhou Medical University, Guangzhou, 510260 China

## Abstract

Angiogenesis is a hallmark of cancer. However, most malignant solid tumors exhibit robust resistance to current anti-angiogenic therapies that primarily target VEGF pathways. Here we report that endothelial-mesenchymal transformation induces glioblastoma (GBM) resistance to anti-angiogenic therapy by downregulating VEGFR-2 expression in tumor-associated endothelial cells (ECs). We show that VEGFR-2 expression is markedly reduced in human and mouse GBM ECs. Transcriptome analysis verifies reduced VEGFR-2 expression in ECs under GBM conditions and shows increased mesenchymal gene expression in these cells. Furthermore, we identify a PDGF/NF-κB/Snail axis that induces mesenchymal transformation and reduces VEGFR-2 expression in ECs. Finally, dual inhibition of VEGFR and PDGFR eliminates tumor-associated ECs and improves animal survival in GBM-bearing mice. Notably, EC-specific knockout of PDGFR-β sensitizes tumors to VEGF-neutralizing treatment. These findings reveal an endothelial plasticity-mediated mechanism that controls anti-angiogenic therapy resistance, and suggest that vascular de-transformation may offer promising opportunities for anti-vascular therapy in cancer.

## Abstract (web summary)

Resistance to anti-angiogenic therapies often occurs in patients. Here, the authors demonstrate the role of PDGF signaling in GBM resistance to anti-VEGF treatment via a mechanism that involves endothelial-mesenchymal transformation and transcriptional regulation of VEGFR-2.

## Introduction

Malignant solid tumors are characterized by excessive and overgrown vasculature^1–3^. The vascular microenvironment fuels tumor growth and progression by supplying oxygen and diffusible nutrients and by releasing soluble factors that promote tumorigenesis^2,4–12^. Therefore, anti-vascular treatment, i.e., eradication and functional inhibition of tumor-associated vascular endothelial cells (ECs), has emerged as a crucial strategy for cancer therapy^3^. However, current anti-angiogenic therapies that primarily target vascular endothelial growth factor (VEGF) pathways have encountered difficulties and failures in treating most malignant cancers. Multiple mechanisms contribute to the tumor resistance to anti-VEGF treatment, including angiogenic pathway redundancy, compensatory activation of survival signals, and pericyte and macrophage-mediated protection^13^. Notably, our recent work reveals robust EC plasticity in tumor microenvironment, e.g., ECs acquire mesenchymal phenotypes to promote their ability to proliferate and migrate^14^, which may alternatively induce primary and acquired resistance to anti-angiogenic treatment in cancer.

Glioblastoma multiforme (GBM), the grade IV glioma, is among the most lethal of human malignancies, distinguished by prominent vascularity. GBM is the most common and most aggressive primary brain tumor in humans, with a current median survival of approximately 14 months^15,16^. Most GBM tumors are refractory to conventional cytotoxic therapies^16^. Anti-angiogenic therapies by VEGF blockade and VEGF receptor inhibition have been exploited in GBM; however, the therapeutic benefits have been small and transient^17–21^. As the major regulator of angiogenesis, VEGF receptor-2 (VEGFR-2) mediates the almost all EC responses to VEGFs, while VEGFR-1 acts as a decoy receptor to modulate VEGFR-2 activity, and VEGFR-3 has a limited role in regulating lymphangiogenesis^22–24^. Here we show that platelet-derived growth factor (PDGF)-mediated endothelial-mesenchymal transformation (Endo-MT) induces EC resistance to anti-angiogenic treatment through downregulation of VEGFR-2 expression. PDGFs are major mitogens for many cell types of mesenchymal origin, including fibroblasts and smooth muscle cells^25,26^. Interestingly, either pharmacological inhibition or genetic deletion of PDGF receptor sensitizes VEGF/VEGFR-2-directed therapy in a mouse GBM model, suggesting that targeting Endo-MT by PDGF inhibition may offer promising opportunities for overcoming anti-VEGF resistance in tumors. Thus, combination of vascular de-transformation with conventional anti-angiogenic treatment may serve as an efficient strategy for anti-vascular therapy in GBM and possibly other malignant solid tumors.

## Results

### Tumor-associated ECs exhibit diminished VEGFR-2 expression

**Fig. 1:**
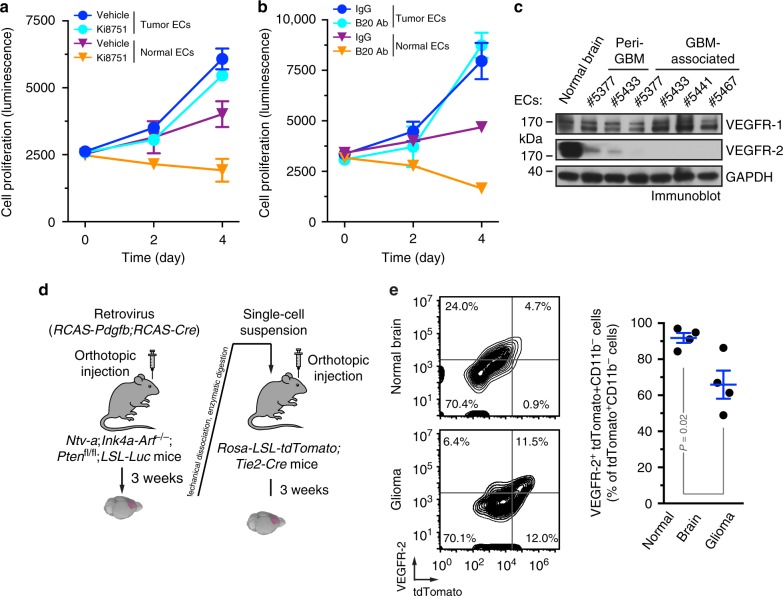
Tumor-associated ECs are resistant to anti-VEGF treatment and have diminished VEGFR-2 expression. **a**–**c** ECs were isolated from GBM tumors or peri-tumor tissues of human patients or normal brains. **a**, **b** Tumor ECs and normal brain microvascular ECs were treated with **a** 3 nM Ki8751 or **b** 10 μg/ml B20 antibody in VEGF-A-containing culture medium, and subjected to cell viability analysis (*n* = 3, mean ± SEM). **c** Cell lysates were immunoblotted. **d**, **e** The primary GBM in *Ntv-a*;*Ink4a-Arf*^−/−^;*Pten*^−/−^;*LSL-Luc* donor mice was induced by RCAS-mediated somatic gene transfer. Single-cell tumor suspension was injected into *Rosa-LSL-tdTomato*;*Tie2-Cre* mice. **d** Schematic approach. **e** Single-cell suspension isolated from normal brains or tumors were analyzed by flow cytometry. Left: representative sorting of CD11b^−^ cells. Right: quantitative data (*n* = 4 mice, mean ± SEM). *P* value was determined by Student’s *t* test

We investigated the treatment responses of GBM-associated ECs to VEGFR inhibition and VEGF blockade. CD31^+^ ECs were isolated from GBM tumors in human patients, and no contamination with other cell types was validated^14^. Almost all of these cells were positive for EC marker von Willebrand factor (vWF) but negative for pericyte marker NG-2 (Supplementary Fig. 1). Cell viability analyses showed that GBM tumor-derived ECs were resistant to pharmacological inhibition of VEGFR (Fig. 1a) and B20 antibody-mediated blockade of VEGF (Fig. 1b). In contrast, VEGFR inhibitor and VEGF-neutralizing antibody completely abolished cell proliferation in normal brain microvascular ECs. Notably, immunoblot analysis of these cells showed that compared to normal ECs, GBM-associated ECs, isolated from either intratumor or peri-tumor tissue, exhibited diminished expression of VEGFR-2, a receptor that mediates almost all of the known cellular responses to VEGF, while expression of VEGFR-1, a receptor that acts as a decoy receptor sequestering VEGF from VEGFR-2 binding, was at similar level in normal and GBM ECs (Fig. 1c), providing a possible mechanism for the anti-VEGF resistance in GBM ECs. Consistently, pharmacological inhibition of VEGFR markedly reduced VEGFR-2^+^ cell population in GBM ECs but not in normal ECs, suggesting more robust anti-VEGF resistance in VEGFR-2^−^ GBM ECs (Supplementary Fig. 2), compared to VEGFR-2^+^ ECs.

To characterize VEGFR-2 expression in tumor-associated ECs in vivo, we took advantage of a genetically engineered murine glioma model, induced by RCAS/N-tva-mediated somatic *Pdgf* gene transfer in *Ink4a-Arf*^−/−^;*Pten*^−/−^ neural stem/progenitor cells (Fig. 1d). Notably, the transgenic GBM mouse model recapitulates the major features of human GBM, including prominent vascularity and vascular abnormality^8,27^. Furthermore, we tested two EC lineage tracing systems, based on Tie2-Cre/Rosa-LSL-tdTomato and Cdh5-Cre^ERT2^/Rosa-LSL-tdTomato, to visualize EC-originated cells in a native glioma microenvironment^28^. Compared with *Cdh5-Cre*^ERT2^;*Rosa-LSL*-*tdTomato* mice, *Tie2-Cre*;*Rosa-LSL*-*tdTomato* mice exhibited much more robust fluorescence in ECs, which therefore is adopted in our flow cytometry analysis (Fig. 1d). Previous work has shown that non-EC myeloid cells, and to a lesser extent, pericytes, also express Tie2^29–31^, which may contribute to the tumor-associated tdTomato^+^ cells derived from Tie2-Cre^+^ lineage. However, our previous work showed that <5% of Tie2^+^ cells expressed myeloid marker CD11b or pericyte marker NG-2 in the mouse GBM tumors, suggesting a minimal contribution of monocytes/macrophages or pericytes to the Tie2^+^ cell population in the GBM model^14^. To exclude the minor population of myeloid cells, we specifically analyzed VEGFR-2 expression in tdTomato^+^CD11b^−^ ECs by flow cytometry. Our data showed that about one-third of tumor-associated ECs lost VEGFR-2 expression in mouse glioma (Fig. 1e), validating the downregulation of VEGFR-2 expression in human GBM ECs. Consistent with these findings, immunofluorescence analysis of surgical tumor specimens from human GBM patients showed diminished expression of VEGFR-2 in a certain part of CD31^+^ vasculatures in the tumors (Supplementary Fig. 3).

### PDGF downregulates VEGFR-2 expression in ECs

**Fig. 2:**
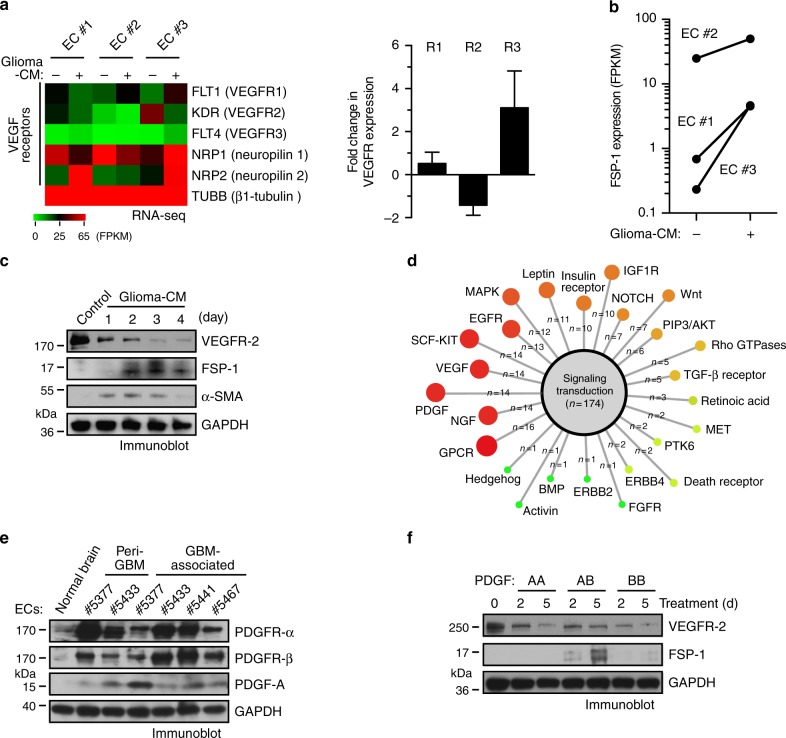
PDGF induces downregulation of VEGFR-2 expression in ECs. **a**, **b** Normal human brain microvascular ECs (#1 and #2 from adult brain and #3 from fetal brain) were treated with glioma-conditioned medium (glioma-CM). RNA was isolated and subjected to transcriptome analysis by RNA deep sequencing (RNA-seq). Left, heat map for expression of VEGF receptors. Right, fold changes of VEGFR-1, VEGFR-2, and VEGFR-3 (*n* = 3, mean ± SEM). **b** Shown are FPKM values of FSP-1 (*n* = 3). **c** Normal brain ECs were treated with glioma-CM or control normal medium. Cell lysates were immunoblotted. **d** Gene set analysis of upregulated pathways/genes identified by RNA-seq in glioma-CM-treated ECs. **e** ECs were isolated from GBM tumors or peri-tumor tissues of human patients or normal brains. Cell lysates were immunoblotted. Note: the lyates were also immunoblotted in Fig. 1c, and the same blot for GAPDH was shown. **f** Normal brain ECs were treated with 100 ng/ml PDGF-AA, PDGF-AB, or PDGF-BB. Cell lysates were immunoblotted

To verify GBM microenvironment-dependent alternation of VEGFR expression, human microvascular ECs, isolated from fetal and adult brain tissues (almost all of these cells were vWF^+^NG-2^−^, Supplementary Fig. 1), were treated with glioma-conditioned medium (glioma-CM, collected from human U251 or primary GBM cells cultured under hypoxia) in vitro. Transcriptome analysis by deep RNA-seq revealed that the treatment downregulated VEGFR-2 expression by about 80%, while increased VEGFR-1 and VEGFR-3 expression (Fig. 2a). Interestingly, RNA-seq also showed that glioma-CM increased expression of mesenchymal marker fibroblast-specific protein (FSP)-1 (Fig. 2b). Immunoblot analysis confirmed these glioma-CM-induced changes in FSP-1 and VEGFR-2 expression, and also showed that glioma-CM induced expression of alpha-smooth muscle actin (α-SMA), another mesenchymal marker protein (Fig. 2c), consistent with our previous work showing that glioma-CM induced mesenchymal transformation in ECs^14^. In addition, similar results were observed in ECs that were treated with glioma-CM harvested from U251 glioma cells cultured under normoxia (Supplementary Fig. 4). Moreover, glioma-CM induced a cell morphology shift from the characteristic cobblestone appearance to fibroblast-like, spindle-shaped cells with disrupted distribution (Supplementary Fig. 5). Interestingly, VEGFR-2^−^ ECs expressed FSP-1 and α-SMA at a higher level and exhibited greater proliferative capacity, compared with those VEGFR-2^+^ ECs isolated from the same human GBM tumor (Supplementary Fig. 6), implicating that loss of VEGFR-2 expression correlates to EC acquisition of mesenchymal transformation and phenotypes including enhanced proliferation.

Furthermore, we explored possible signaling activation in glioma-CM-treated ECs by gene set enrichment analysis of the upregulated genes identified by RNA-seq. Top three identified pathways included G-protein coupled receptor (GPCR), nerve growth factor (NGF), and PDGF signaling pathways (Fig. 2d). Considering (i) the well-established role of PDGF for functional regulation in mesenchymal cells and (ii) no specific, defined role of GPCR and NGF in endothelial biology, we focused our study on the PDGF pathway. Immunoblot analysis of ECs derived from normal brain, peri-tumor tissues, and tumors showed that GBM-associated ECs robustly expressed PDGFR-α, PDGFR-β, and PDGF-A (Fig. 2e). We next tested if PDGFs, including AA, AB, and BB dimers, could modulate mesenchymalization and VEGFR-2 expression in ECs. Notably, all PDGFs markedly reduced VEGFR-2 expression, while only PDGF-AB robustly induced expression of mesenchymal gene *FSP-1* (Fig. 2f). Together, these results suggest that PDGF may regulate mesenchymalization and VEGFR-2 expression in ECs.

### PDGF is critical for glioma-CM-reduced VEGFR-2 expression

**Fig. 3:**
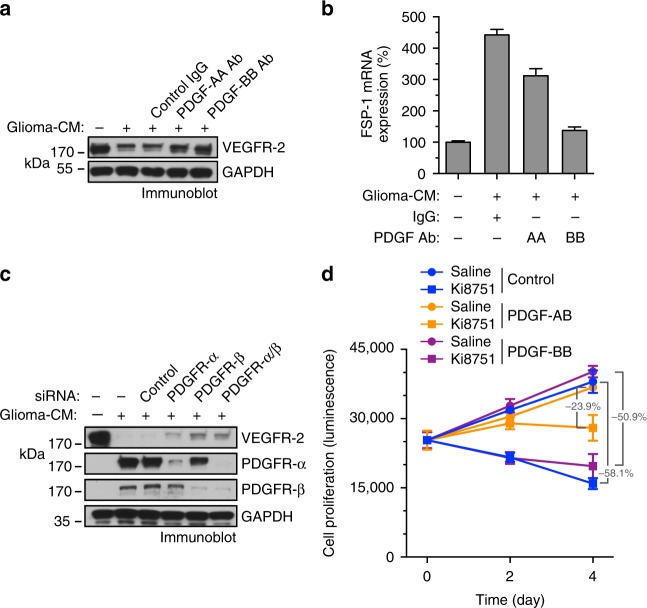
PDGF is critical for glioma-CM-induced VEGFR-2 down-expression and EC resistance to anti-VEGF treatment. **a**, **b** Human brain microvascular ECs were treated with glioma-CM in the absence or presence of anti-PDGF-AA or anti-PDGF-BB neutralizing antibody or control IgG. **a** Cell lysates were immunoblotted. **b** RNA was isolated and analyzed by RT-PCR. Results were normalized with GAPDH levels (*n* = 3–5, mean ± SD). **c** Normal brain ECs were transfected with siRNAs targeting PDGFR-α, PDGFR-β, or control scrambled sequence, and treated with glioma-CM. Cell lysates were immunoblotted. **d** Human brain ECs were pretreated with 100 ng/ml PDGF-AB or PDGF-BB, followed by treatment with 3 nM Ki8751. Cell proliferation was determined (*n* = 3, mean ± SD). *P* values were determined by Student’s *t* test

We investigated the role of PDGF in VEGFR-2 down-expression and anti-VEGF treatment resistance in ECs under glioma conditions. Our data showed that neutralization of PDGF by using either anti-PDGF-AA or anti-PDGF-BB antibody restored VEGFR-2 expression (Fig. 3a) and inhibited FSP-1 expression (Fig. 3b) in glioma-CM-treated ECs. Moreover, knockdown of PDGFR-α, and to a greater extent, PDGFR-β, induced re-expression of VEGFR-2 expression in glioma-CM-treated ECs (Fig. 3c), suggesting that PDGF/PDGFR is critical for glioma-CM-induced VEGFR-2 down-expression in ECs. Furthermore, pretreatment of ECs with PDGF-AB, but not with PDGF-BB, render cells to resist to pharmacological VEGF inhibition with Ki8751 (Fig. 3d). These findings establish a critical in vitro role of PDGF-AB for VEGFR-2 down-expression and anti-VEGF resistance in ECs under GBM conditions.

### PDGF induces Endo-MT through nuclear factor-κB-mediated Snail expression

**Fig. 4:**
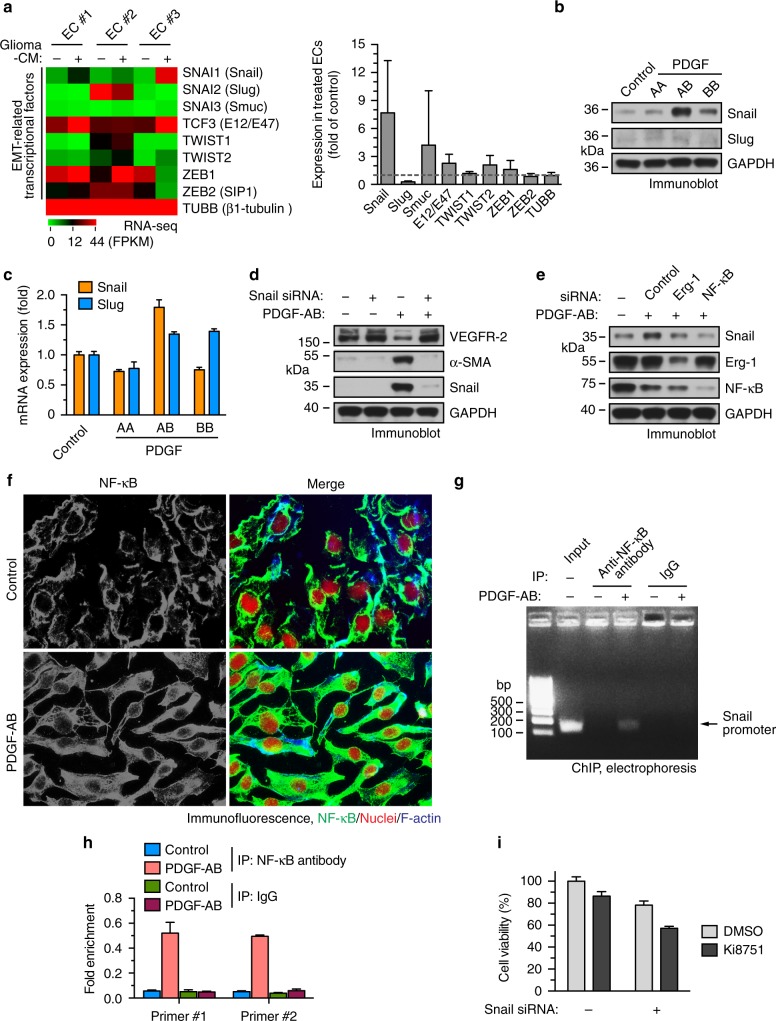
PDGF-AB induces Endo-MT through NF-kB-mediated Snail expression in ECs. **a** Human brain microvacular ECs were treated with glioma-CM. RNA was isolated and subjected to RNA-seq analysis. Left, heat map for expression of EMT-related transcriptional factors. Right, fold change of these transcriptional factors (*n* = 3, mean ± SEM). **b**, **c** ECs were treated with 100 ng/ml PDGF-AA, PDGF-AB, and PDGF-BB. **b** Cell lysates were immunoblotted. **c** RNA was isolated and analyzed by RT-PCR. Results were normalized with GAPDH levels (*n* = 3, mean ± SEM). **d** ECs were transfected with siRNAs targeting Snail or control scrambled sequence, and treated with PDGF-AB. Cell lysates were immunoblotted. **e** ECs were transfected with siRNAs targeting Erg-1, NF-κB, or control scrambled sequence, and treated with PDGF-AB. Cell lysates were immunoblotted. **f** ECs were treated with PDGF-AB for 2 h. Cells were analyzed by immunofluorescence. **g**, **h** ECs were treated with PDGF-AB or control medium for 8 h. Nuclear extracts were immunoprecipitated with anti-NF-κB antibody or IgG, and subjected to ChIP analysis with primers #1 and #2. **g** DNA was resolved by agarose electrophoresis, and imaged. Shown are representative results with primer #2. The arrow indicates the amplified DNA in Snail promoter. **h** Quantitative PCR analysis (*n* = 3, mean ± SEM). **i** ECs were transfected with siRNAs targeting Snail or control scrambled sequence, 4 days after treatment with Ki8751, followed by cell viability analysis (*n* = 3, mean ± SD)

We then explored the molecular mechanism(s) underlying Endo-MT, initially focusing on the transcriptional factors that control epithelial-mesenchymal transition (EMT), which include Snail, Slug, Smuc, Tcf3, Twist1/2, and Zeb1/2. RNA-seq analysis revealed that glioma-CM induced a robust increase in expression of Snail, but not on other transcriptional factors (Fig. 4a). Interestingly, treatment of normal brain ECs with PDGF-AB, and to a less extent, PDGF-BB, markedly stimulated protein expression of Snail (Fig. 4b). Similarly, PDGF-AB increased Snail mRNA expression (Fig. 4c), collectively suggesting that PDGF-AB may induce Endo-MT through Snail. Consistent with this hypothesis, small interfering RNA (siRNA)-mediated knockdown of Snail restored VEGFR-2 expression and abrogated α-SMA expression in PDGF-AB-treated ECs (Fig. 4d). Furthermore, chromatin-immunoprecipitation (ChIP) analysis showed that PDGF-AB induced Snail binding to VEGFR-2 promoter in ECs (Supplementary Fig. 7), suggesting a critical role for Snail in PDGF-induced Endo-MT and VEGFR-2 down-expression.

To gain a molecular insight into the transcriptional regulation mechanism of Snail, we analyzed its promoter sequence and predicted that the transcriptional factors, including AREB6, AP-1, c-Jun, Erg-1, and nuclear factor-κB (NF-κB) may possibly bind to the region based on motif recognition pattern. Among this list of transcriptional regulators, Erg-1 and NF-κB are known to be activated by PDGF^32,33^. Our data revealed that NF-κB siRNA almost completely abolished PDGF-induced snail expression, while Erg-1 siRNA only slightly attenuated snail expression (Fig. 4e), suggesting that NF-κB is critical for PDGF-AB-induced snail expression. Moreover, immunofluorescence analysis verified PDGF-induced NF-κB activation in brain ECs, as indicated by their translocation to nuclei (Fig. 4f). Furthermore, ChIP analysis showed that PDGF-AB induced NF-κB binding to snail promoter (Fig. 4g, h). Finally, siRNA-mediated knockdown of Snail sensitized GBM ECs to pharmacological inhibition of VEGFR-2 (Fig. 4i). Taken together, these findings suggest that PDGF induces Endo-MT and VEGFR-2 down-expression through NF-κB-mediated Snail expression.

### PDGF autocrine downregulates VEGFR-2 expression in GBM ECs

**Fig. 5:**
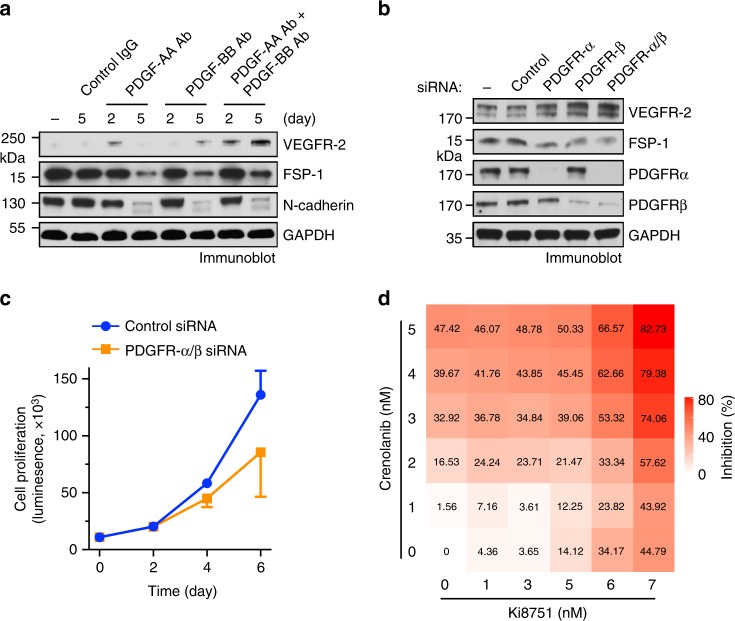
PDGF autocrine loop is critical for VEGFR-2 down-expression and anti-VEGF resistance in GBM-associated ECs. **a** GBM tumor-derived ECs were treated with control IgG or antibody against PDGF-AA or PDGF-BB. Cell lysates were immunoblotted. **b**, **c** GBM tumor-derived ECs were transfected with control scrambled siRNA or siRNA targeting PDGFR-α and PDGFR-β. **b** Cell lysates were immunoblotted. **c** Cell proliferation was determined (*n* = 3, mean ± SEM). **d** GBM tumor-derived ECs were treated with Ki8751 and crenolanib at different doses. Cell proliferation was determined 4 days after treatment. Inhibition rates were calculated and expressed as % of control cells

GBM-associated ECs robustly expressed both PDGFRs and PDGF (Fig. 2d), suggesting a possible autocrine mechanism that regulates EC mesenchymalization and VEGFR-2 down-expression. To test this hypothesis, GBM-derived ECs were treated with PDGF-neutralizing antibodies. Immunoblot analysis showed that PDGF neutralization inhibited mesenchymalization in GBM-associated ECs, as evidenced by that 5-day incubation with anti-PDGF-AA antibody, anti-PDGF-BB antibody, alone or combined, abolished FSP-1 and N-cadherin expression (Fig. 5a). Moreover, co-treatment with anti-PDGF-AA and anti-PDGF-BB antibodies enhanced VEGFR-2 expression in GBM ECs (Fig. 5a). Similarly, siRNA-mediated knockdown of PDGFR-α and PDGFR-β significantly attenuated FSP-1 expression and increased VEGFR-2 expression (Fig. 5b) and inhibited cell proliferation (Fig. 5c), suggesting a requisite role of autocrined PDGF/PDGFR for maintenance of mesenchymalization, VEGFR-2 down-expression, and hyper-proliferation in GBM ECs. Furthermore, co-treatment of tumor ECs with VEGFR-2 inhibitor Ki8751 (IC_50_ = 0.9 nM) and PDGFR inhibitor crenolanib (IC_50_ = 0.9 and 1.8 nM for PDGFR-α and PDGFR-β, respectively) inhibited EC viability in a dose-dependent manner (Fig. 5d), suggesting that PDGFR inhibition sensitizes GBM ECs to anti-VEGF treatment.

### Dual inhibition of PDGFR and VEGFR-2 abrogates glioma growth

**Fig. 6:**
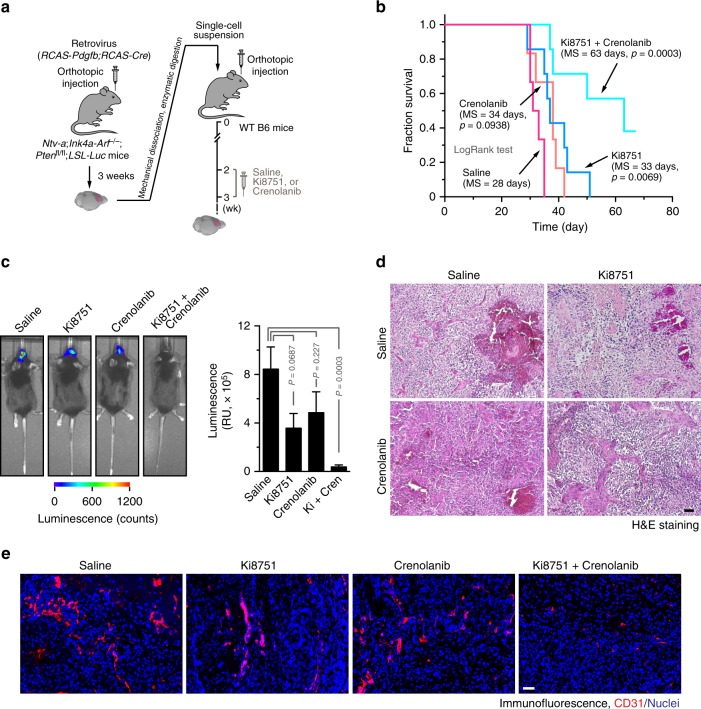
Dual inhibition of PDGFR and VEGFR-2 inhibits glioma growth and progression. The primary GBM was induced in *Ntv-a*;*Ink4a-Arf*^−/−^;*Pten*^−/−^;*LSL-Luc* donor mice by RCAS-mediated somatic gene transfer. Single-cell tumor suspension was implanted into recipient mice, followed by treatment. **a** Schematic approach. **b** Animal survival was monitored for 70 days after injection (*n* = 6–7 mice). MS median survival. *P* values were determined by log-rank tests. **c** Tumor growth was analyzed by whole-body bioluminescence imaging. Left, representative images. Right, quantitative analysis of integrated luminescence in tumors at day 32–38 (*n* = 3–6, mean ± SEM). *P* values were determined by Student’s *t* test. **d** Tumor sections were stained with H&E and imaged. Representative data are shown from 3–4 mice/group. Scale bar: 100 μm. **e** Tumor sections were immunostained with anti-CD31 antibody and imaged. Shown are representative images (*n* = 3–5 mice). Scale bar: 100 μm

Based on the synergic effects of dual inhibition of PDGFR and VEGFR on cell proliferation in GBM ECs in vitro, we next investigated its effects on tumor vascularization and growth in vivo. The RCAS transgenic GBM model was orthotopically induced in mice, followed by single or combined treatment with Ki8751 and crenolanib (Fig. 6a). Our data showed that single crenolanib treatment did not significantly affect mouse survival (Fig. 6b) or tumor growth (Fig. 6c). Similarly, single Ki8751 treatment slightly increased mouse survival (+6 days) but did not significantly inhibit tumor growth. However, dual inhibition markedly enhanced mouse survival (+35 days) and robustly abrogated tumor growth. Notably, almost half of the mice in the dual inhibition group still survived when all mice in other three groups died 42 days after tumor induction, and over 20% of dual treatment mice survived for at least 70 days when the experiment was terminated (Fig. 6b). Moreover, dual inhibition substantially inhibited tumor growth, as evidenced by a significant decrease in tumor volume (Fig. 6c). These results indicate that PDGFR inhibition robustly sensitizes VEGFR-inhibiting treatment in GBM.

Tumor vessels of control saline-treated mice exhibited typical morphological features of high vascularity with extensive hemorrhage (Fig. 6d). Single treatment with Ki8751 or crenolanib did not alter pathological appearance (Fig. 6d) or significantly block tumor vascularization (Fig. 6e). However, combined treatment with Ki8751 or crenolanib inhibited hemorrhagic necrosis, a defining pathological feature of GBM (Fig. 6d), and more importantly, significantly reduced vascular density in the tumors (Fig. 6e). These results suggest that dual inhibition of PDGFR and VEGFR may suppress GBM growth by inhibiting tumor vascularization.

### PDGFR-β deletion in ECs sensitizes anti-VEGF therapy

**Fig. 7:**
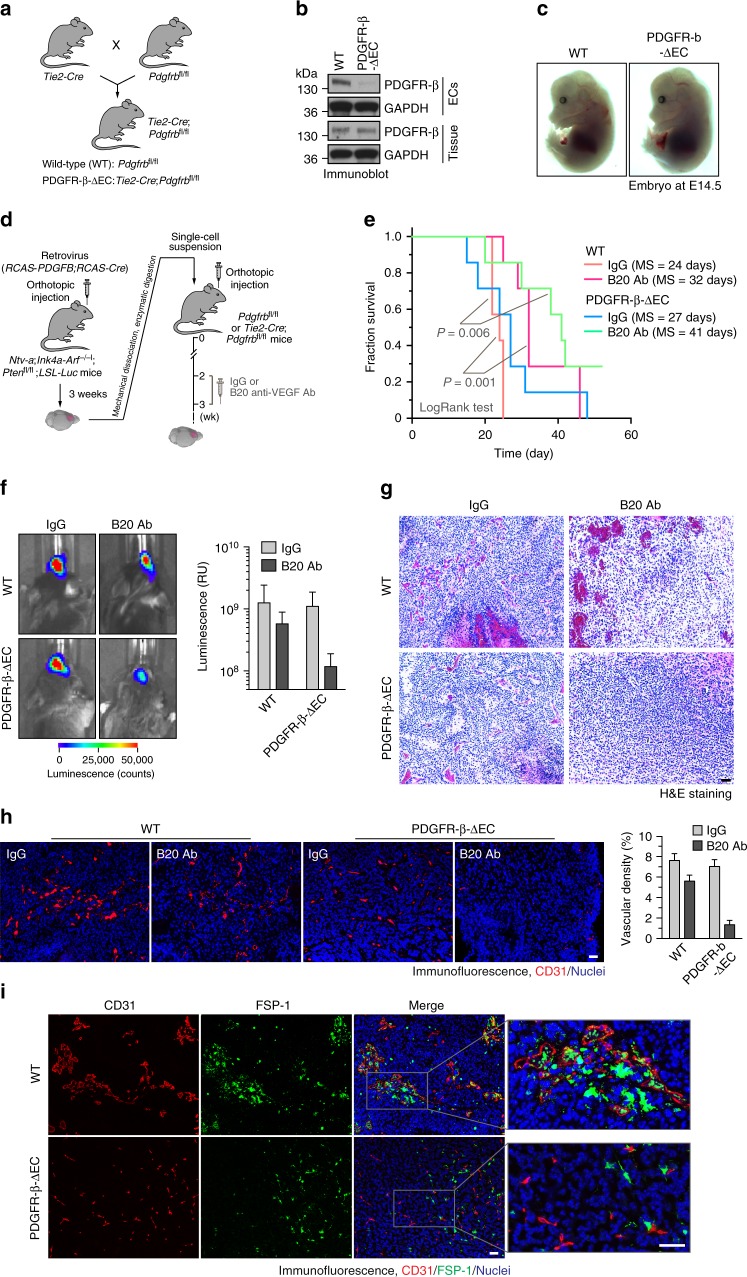
Endothelial-specific deletion of PDGFR-b sensitizes glioma-associated ECs and tumors to anti-VEGF treatment. **a**–**c** *Tie2-Cre;Pdgfrb*^fl/fl^ mice were generated by crossing *Tie2-Cre* mice with *Pdgfrb*^fl/fl^ mice. **a** Schematic approach. **b** ECs were isolated from mouse aortas. Heart tissue and ECs were subjected to immunoblot analysis. **c** Mouse embryos were imaged (*n* = 5). **d**–**h** The primary GBM in *Ntv-a*;*Ink4a-Arf*^−/−^;*Pten*^−/−^;*LSL-Luc* donor mice was induced by RCAS-mediated somatic gene transfer. Single-cell tumor suspension was implanted into *Pdgfrb*^fl/fl^ (WT) or *Tie2-Cre*;*Pdgfrb*^fl/fl^ (PDGFR-β-ΔEC) recipient mice, followed by treatment with B20 antibody and IgG. **d** Schematic approach. **e** Animal survival was monitored for 60 days after injection (*n* = 7 mice). MS median survival. *P* values were determined by log-rank tests. **f** Tumor growth was analyzed by whole-body bioluminescence imaging. Left, representative images. Right, quantitative analysis of integrated luminescence in tumors at day 25–28 (*n* = 3–6 mice, mean ± SEM). **g** Tumor sections were stained with H&E dyes. Representative data are shown (*n* = 3–4 mice). Scale bar: 100 μm. **h** Tumor sections were immunostained with anti-CD31 antibody. Left, representative images. Right, quantitative analysis of CD31 fluorescence area (*n* = 4–5 mice, mean ± SEM). Scale bar: 100 μm. **i** Tumor sections were immunostained with anti-CD31 and anti-FSP-1 antibodies. Representative images are shown (*n* = 4–5 mice). Scale bar: 100 μm

To rigorously determine if the antitumor effects of dual inhibition act mainly through its effects on tumor-associated ECs, we generated an EC-specific PDGFR-β-knockout mouse line, *Tie2-Cre*;*Pdgfrb*^fl/fl^ by crossing *Pdgfrb*^fl/fl^ mice with mice expressing Cre under EC-specific promoter *Tie2* (Fig. 7a). PDGFR-β was selected to be targeted because of its well-established developmental role for blood vessel formation and early hematopoiesis, while PDGFR-α signaling is critical for gastrulation and the development of the cranial and cardiac neural crest, gonads, and other organs^25^. EC-specific PDGFR-β knockout was verified by immunoblot (Fig. 7b). Interestingly, *Pdgfrb* deletion in ECs did not affect basal angiogenesis, as indicated by apparently normal embryos (Fig. 7c), suggesting a dispensable role of endothelial PDGFR-β in developmental angiogenesis and normal tissue functions.

We then challenged these mice with an orthotopic injection of the tumor cells isolated from the RCAS transgenic GBM model (Fig. 7d). Our results showed that EC-specific PDGFR-β knockout did not affect animal survival (Fig. 7e). In the control *Pdgfrb*^fl/fl^ mice (refer to wild-type (WT) mice), B20 anti-VEGF antibody moderately improved animal survival (+9 days, Fig. 7e). However, EC-specific PDGFR-β knockout remarkably sensitized GBM tumors to anti-VEGF treatment, as indicated by improved (+17 days) median survival in *Tie2-Cre;Pdgfrb*^fl/fl^ mice (Fig. 7e). Notably, about 30% of PDGFR-β-knockout mice survived for at least 50 days after the experiment was terminated, further suggesting the significance of this EC resistance-mediated tumorigenesis. Consistently, B20 antibody treatment reduced tumor growth in PDGFR-β-knockout mice, while there was a slight effect on control WT mice (Fig. 7f). In addition, considering that pericytes also express Tie2 at a lower level^31^, we tested if Tie2 promoter-mediated Cre expression induces PDGFR-β deletion in pericytes, which, in turn, may affect pericyte survival/growth in tumors. Immunofluorescence analysis shows that the tumors in *Pdgfrb*^fl/fl^ and *Tie2-Cre*;*Pdgfrb*^fl/fl^ mice exhibited similar patterns of NG-2^+^ pericyte distribution and PDGFR-β expression (Supplementary Fig. 8), verifying the EC specificity of Tie2-Cre system in these tumor models.

Furthermore, B20 antibody treatment did not affect tumor pathological appearance in WT mice, but inhibited hemorrhagic necrosis in the PDGFR-β-knockout mice (Fig. 7g). Similarly, B20 antibody treatment slightly reduced vascular density in WT mice, but markedly abrogated tumor vascularization in the PDGFR-β-knockout mice (Fig. 7h). In addition, tumor vessels of control WT mice exhibited typical morphological features of vascular abnormality that is common in human GBM, i.e., they are tortuous and dilated with extensive hemorrhage. Interestingly, the blood vessels in the PDGFR-β-knockout mice appeared partially normalized, as evidenced by non-tortuous vessels (Fig. 7h, i) with minimal hemorrhage and necrosis (Fig. 7g). Consistent with the role of PDGFR-β in Endo-MT, PDGFR-β knockout in ECs reduced FSP-1 expression in CD31^+^ ECs (Fig. 7i). These findings suggesting that PDGFR-β knockout inhibits Endo-MT and sensitizes tumor ECs to anti-VEGF treatment.

**Fig. 8:**
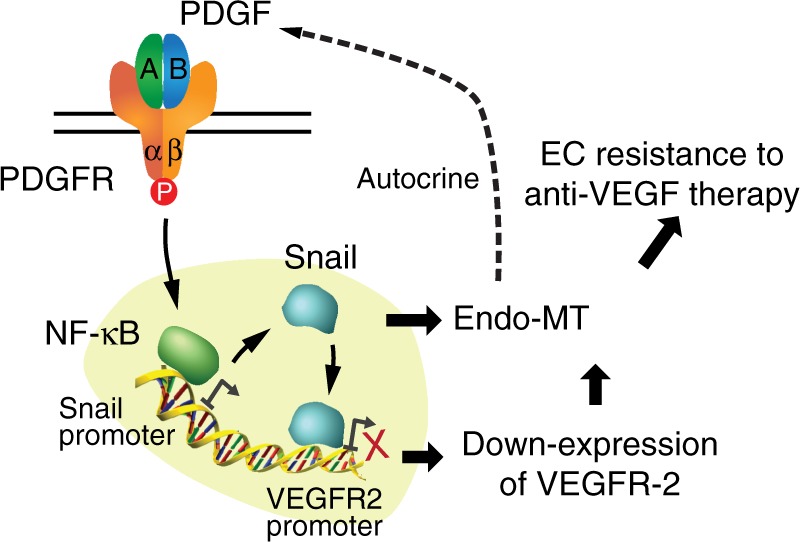
A schematic model. PDGF in the tumor microenvironment activates PDGF receptor in ECs, which in turn induces NF-κB-dependent Snail expression, thereby inducing endothelial-mesenchymal transformation (Endo-MT). Snail binds to VEGFR-2 promoter and suppresses VEGFR-2 transcription, resulting in VEGFR-2 down-expression and Endo-MT, and eventually leading to anti-VEGF resistance in ECs. In addition, Endo-MT stimulates PDGF expression in ECs, potentially serving as an autocrine-mediated positive forward feedback loop that drives Endo-MT and EC resistance to anti-VEGF treatment

Collectively, we show that Endo-MT and VEGFR-2 down-expression regulates EC resistance to anti-VEGF treatment (Fig. 8). We identify a PDGF/NF-κB/Snail axis that controls Endo-MT and VEGFR-2 down-expression. In addition, Endo-MT stimulates PDGF expression in ECs, which may serve as an autocrine-mediated positive feedback loop.

## Discussion

Angiogenesis is a hallmark of solid tumor development and progression^3^. However, current anti-angiogenic therapy that primarily targets VEGF/VEGFR-2 has encountered difficulties and failures in treating most malignant solid tumors, including GBM^17–21^, likely due to insufficient eradication and functional inhibition of tumor-associated ECs. Endo-MT may contribute to both intrinsic and acquired tumor resistance to anti-VEGF therapy, by VEGFR-2 downregulation-mediated treatment unresponsiveness and by selective regrowth of highly proliferative, transformed ECs after treatment, respectively. We propose vascular de-transformation as a novel strategy for anti-vascular therapy in cancer. Our study reveals that Endo-MT abrogates VEGFR-2 expression in a certain population of GBM-associated ECs. These ECs may acquire mesenchymal transformation and genetic reprograming, bypassing angiogenic pathways to support their survival, proliferation, and migration in the tumor microenvironment, and to evade from the cytotoxic effects induced by conventional anti-vascular therapies that target pro-angiogenic factors. Likewise, our results show that human GBM-derived primary ECs exhibit robust resistance to anti-VEGF/VEGFR-2 treatment, challenging VEGF as the primary, sole target for anti-vascular cancer therapy.

Our previous work characterizes robust EC plasticity in GBM microenvironment: ECs undergo Endo-MT to promote their ability to proliferate and migrate, leading to c-Met-mediated vascular abnormality and chemoresistance^14^. Moreover, Endo-MT suggests that no cell fate transition occurs and key endothelial functions are retained, which is distinguished from previously proposed endothelial-mesenchymal transition that generates tumor-associated fibroblasts de novo^14,34^. Here our unbiased transcriptome analysis by RNA-seq validates mesenchymal transformation in the ECs under tumor conditions, and importantly, reveals VEGFR-2 down-expression. The loss of VEGFR-2 expression indicates that the transformed ECs have developed a VEGF-independent mechanism to maintain EC growth and survival in GBM, rendering EC resistance to anti-VEGF treatment. On the other hand, GBM ECs acquire mesenchymal phenotypes, including enhanced proliferation^14^, leading to overgrowth of these treatment-resistant, transformed ECs in the tumor microenvironment. In addition, glioma stem cells express VEGFR-2 and form vasculogenic mimicry, as an alternative mechanism for GBM vascularization^35–37^. Recent studies show that bevacizumab does not affect glioma stem cell-mediated vasculogenic mimicry^38,39^, suggesting that this mechanism may also contribute to anti-VEGF resistance in GBM. We suggest that complete eradication of GBM vasculature may need to target both tumor-associated ECs and glioma stem cells. Notably, our RNA-seq analysis identifies PDGF as a critical regulator of Endo-MT, which reduces VEGFR-2 expression in ECs. PDGF signaling has been well recognized to be activated in mesenchymal cells, including fibroblasts, smooth muscle cells, and pericytes^25,40^. Interestingly, our data suggest that mesenchymal transformation renders tumor-associated ECs to upregulate PDGF/PDGFR expression. Moreover, normal development of mice with EC-specific PDGFR-β deficiency indicates a dispensable role of endothelial PDGF signaling for physiological functions, suggesting PDGF as a selective and safe target for anti-vascular therapy.

A previous report shows that pharmacological PDGFR inhibition sensitizes mouse pancreatic cancer to VEGFR-2-targeting treatment, which could be explained by that PDGFR inhibition eradicates pericytes that protect ECs against pharmacological inhibition of VEGFR-2^41^. Moreover, a recent clinical study shows a greater therapeutic efficacy by dual antagonism of PDGF and VEGF in treating age-related macular degeneration^42^. Here, by utilizing specific pharmacological inhibitors, we consistently show that dual inhibition of PDGFR and VEGFR-2 efficiently reduces GBM vascularization and exhibits greater antitumor activity. However, our further results show that EC-specific deletion of PDGFR-β sensitizes VEGF blockade treatment, and the anti-VEGF antibody efficiently eradicates ECs in the PDGFR-β-knockout mice but not in WT mice, suggesting that PDGF inhibition mainly acts on ECs to overcome their anti-VEGF resistance. In addition, more profound effects by dual pharmacological inhibition (Fig. 6) than VEGF neutralization plus EC-specific PDGFR-β knockout (Fig. 7) in our animal studies implicate that pericyte eradication by PDGF inhibition may exert additional benefits in GBM therapy. Nevertheless, the loss of pericytes that results from blocking PDGFRs may destabilize tumor vessels, which, in turn, impairs drug delivery and creates a hostile microenvironment to promote cancer progression^43,44^. Therefore, precisely targeting EC transformation may present a promising strategy for cancer therapy.

The regulatory mechanisms underlying Endo-MT remain largely unknown. Endothelial plasticity could be mediated through transforming growth factor-beta (TGF-β), bone morphogenic protein, HGF/c-Met, and Notch pathways^14,45,46^. Here we identify Snail as a key downstream regulator of Endo-MT in cancer settings. We reveal that PDGF induces NF-κB-dependent Snail expression, leading to Endo-MT and VEGFR-2 down-expression. Snail plays a key role for EMT in tumor cells^47–49^. NF-κB contributes to regulation of Snail expression during EMT^50,51^. Recent studies suggest that Snail is also critical for endothelial plasticity induced by hypoxia, TGF-β treatment, or shear stress^52–55^. Our work reveals that PDGF-AB induces NF-κB-mediated snail expression in ECs. Snail binds to the promoter region of cadherins, suppressing their transcription and facilitating EMT in epithelial cells^56,57^. Similarly, Snail acts as a transcriptional suppressor of VE-cadherin in ECs^58,59^, which likely contributes to Snail-mediated Endo-MT in cancer.

In summary, our study reveals that mesenchymal transformation drives GBM resistance to anti-angiogenic therapy. We identify a PDGF/NF-κB/Snail axis that controls Endo-MT and VEGFR-2 down-expression. Notably, pharmacological inhibition or genetic ablation of PDGF signaling robustly sensitizes anti-VEGF/VEGFR treatment in GBM. As such, combination of vascular de-transformation with conventional anti-angiogenic therapy may offer exciting opportunities to treat malignant cancer.

## Methods

### EC isolation from patient tumors

Patient samples were collected at the Department of Neurosurgery of the Hospital of the University of Pennsylvania. The collection of human tissues in compliance with the tissue banking protocol was approved by the University of Pennsylvania Institutional Review Board, and written informed consent was obtained from each participant. ECs were isolated and verified^14^. In brief, tumor-derived single-cell suspensions were prepared by the tissue bank. Red cells were removed with ACK lysis buffer (Life Technologies). Cell suspension was subjected to magnetic-activating cell sorting (MACS) with anti-CD31 antibody-conjugated magnetic beads (Miltenyi Biotech, 130-091-935). Sorted ECs were verified by Dil-Ac-LDL (Alfa Aesar, J65597) absorption, and 99% of cells were Dil-Ac-LDL-positive. All cells were checked and showed no mycoplasma contamination.

### Mice

*Tie2-Cre*;*Rosa-LSL-tdTomato* mice were generated by crossing *Rosa-LSL-tdTomato* mice (Jackson Lab) with *Tie2-Cre* mice (Jackson Lab). *Tie2-Cre;Pdgfrb*^fl/fl^ and *Pdgfrb*^fl/fl^ mice were generated by crossing *Pdgfrb*^fl/fl^ mice (Jackson Lab) with *Tie2-Cre* mice (Jackson Lab). *Cdh5-Cre*^ERT2^ mice were generated by Ralf Adams (Max Planck) and kindly provided by Bisen Ding (Cornell)^60^. *Cdh5-Cre*^ERT2^;*Rosa-LSL-tdTomato* mice were generated by crossing *Rosa-LSL-tdTomato* mice (Jackson Lab) with *Cdh5-Cre*^ERT2^ mice. All animals were housed in the Association for the Assessment and Accreditation of Laboratory Animal Care-accredited animal facility of the University of Pennsylvania. All experiments with mice were performed in accordance with a protocol approved by the Institutional Animal Care and Use Committee at the University of Pennsylvania.

### Isolation and culture of mouse ECs

Mouse aorta ECs were isolated and cultured^14^. In brief, thoracic aorta was isolated from 3-week-old *Tie2-Cre;Pdgfrb*^fl/fl^ and *Pdgfrb*^fl/fl^ mice. Aortic rings were embedded in Matrigel-coated dishes and cultured for 5 days. After rinsing with phosphate-buffered saline (PBS), the rings were removed, and remaining cells incubated with 2 U/ml Dispase I (Gibco, 17105-041) for 20 min at 37 °C. After centrifugation at 500 × *g* for 10 min, the cell pellets were washed with PBS, and cells cultured in Dulbecco’s modified Eagle’s medium (DMEM)/F-12 medium supplemented with 25 mg/ml EC growth supplement (Sigma) and 5% fetal bovine serum (FBS) at 37 °C in a humidified air atmosphere with 5% CO_2_.

### Tissue protein extraction

The heart tissues from *Tie2-Cre;Pdgfrb*^fl/fl^ and *Pdgfrb*^fl/fl^ mice were collected and homogenized in PBS with proteinase inhibitor cocktail (Roche, 11697498001), followed by tissue lysis with NP-40 buffer.

### GBM mouse model

GBM was induced in mice^14,27,61,62^. In brief, chicken DF-1 fibroblasts (American Type Culture Collection) were transfected with RCAS-PDGF-B and RCAS-Cre plasmids to produce retrovirus, and orthotopically injected into *Ntv-a*;*Ink4a-Arf*^−/−^;*Pten*^fl/fl^*;LSL-luc* mice to induce GBM through RCAS/n-tva-mediated gene transfer. Tumors were isolated and subjected to mechanical dissociation with a gentle MACS Dissociator (Miltenyi) and enzymatic digestion with collagenase II and dispase to obtain single-cell suspensions. About 8-week-old mice (half male and half female) were orthotopically, stereotactically injected with 10^5^ GBM tumor cells. Tumor growth was monitored by whole-body bioluminescence using an IVIS 200 Spectrum Imaging System after retro-orbital injection of luciferin (150 mg/kg, GoldBio). For tumor induction in *Tie2-Cre;Pdgfrb*^fl/fl^ mice, tumor-derived single-cell suspension was cultured with mouse stem cell medium. Attached cells were discarded to remove tumor stromal cells, and non-adherent sphere-forming tumor cells were injected into mice. Mice were administrated with peritoneal injection of saline, 50 mg/kg Ki8751 or crenolanib, or 10 mg/kg B20 anti-VEGF-A antibody (Roche/Genentech) 2 weeks after GBM induction. Post-injection survival was monitored for 60 days. Mice were euthanized when exhibiting severe GBM symptoms, including domehead, hemiparesis, or more than 20% of body weight loss. Mice were randomized to receive treatment, and the investigators were not blinded.

### MACS for EC separation

GBM ECs were incubated with a biotinylated anti-VEGFR-2 antibody (1:11, Miltenyi Biotech, 130-100-308) for 10 min at 4 °C, followed by incubation with streptavidin-conjugated MicroBeads (Miltenyi Biotech, 120-000-287) for 15 min at 4 °C. Cell suspensions were loaded into a column with a MACS Separator, and then eluted with washing medium. The retained cells and eluted cells were collected.

### Flow cytometry

GBM was induced in *Ntv-a*;*Ink4a-Arf*^−/−^;*Pten*^fl/fl^;*LSL-luc* mice through RCAS/n-tva-mediated gene transfer, followed by implantation into *Tie2-Cre*;*Rosa-LSL-tdTomato* mice. Mouse tumor tissues were obtained, and single-cell tumor suspension was stained with control IgG (Santa Cruz, sc-2025), anti-VEGFR-2 (BioLegend, 136407), and anti-CD11b (BioLegend, 101228) antibodies. After treatment, normal human brain ECs or GBM ECs were stained with control (eBioscience, 17-4724-41), anti-VEGFR-2 (1:100, BioLegend, 359916), anti-NG-2 (1:100, Millipore, AB5320), or anti-vWF (1:100, DAKO, A0082) antibody. The cells were subjected to flow cytometry analysis with an Accuri C6 flow cytometer (BD Biosciences). The data were analyzed using FlowJo software.

### Preparation of glioma cell-CM

Human U251 glioma cells (Sigma) and primary patient glioma cells were cultured with DMEM medium (Invitrogen) supplemented with 5% FBS (Invitrogen)^14^. In brief, when cultures reached more than 90% confluence, cells were exposed to hypoxia (1% O_2_) or normoxia for 24 h. Culture medium was centrifuged at 5000 × *g* for 30 min to remove cellular debris, and the supernatant was collected.

### Cell culture and treatment

Human brain microvascular ECs (ScienCell and PromoCell, isolated from adult or fetal human brains) were maintained in EC medium (ScienCell, supplemented with VEGF-A). All cells were used between passages 2 and 5. All cells were checked and showed no mycoplasma contamination. Cells were treated with recombinant human PDGF-AA (100 ng/ml, Peprotech, 100-13 A), PDGF-AB (100 ng/ml, Peprotech, 100-00AB), PDGF-BB (100 ng/ml, Peprotech, 100-14B), Ki8751 (3 nM, Bio-Techne, 228559-41-9), crenolanib (5 nM, ChemieTek, CP-868596), anti-VEGF antibody B20 (10 μg/ml, Genentech), anti-PDGF-AA (10 μg/ml, Millipore, 07-1436), anti-PDGF-BB (10 μg/ml, Millipore, 07-1437), or control rabbit IgG (10 µg/ml, Cell Signaling, 2729).

### RNA deep sequencing analysis

Human brain microvascular ECs were treated with glioma-CM for 2 days. After changing medium, cells were cultured in DMEM/F-12 medium for 1 day. Approximately 2 × 10^6^ cells were lysed in 1 ml TRIzol (Thermo Fisher), followed by RNA extraction according to the manufacturer’s instructions. The isolated RNA was purified by using an RNeasy Plus Mini Kit (Qiagen). After a quality control step using RNA Nano assay chips with a 2100 bioanalyer (Agilent), library was constructed by using a TruSeq protocol (Agilent), and subjected to deep sequencing (100 PE, about 10 megabyte reads for each sample, Illumina sequencer 2500) at the Children’s Hospital of Philadelphia/Beijing Genomics Institute core facility. The sequences were aligned to the UCSC hg38 reference genome using RNA-Star (v2.4.2a) (https://github.com/alexdobin/STAR). The gene expression was normalized and calculated as fragments per kilobase million values by Cufflinks (v2.2.1) (http://cole-trapnell-lab.github.io/cufflinks/releases/v2.2.1/) with Gencode v22 gene annotations (https://www.gencodegenes.org/releases/22.html).

### Immunofluorescence and histology

Patient samples were collected at the Department of Neurosurgery of the Sun Yat-sen University Cancer Center^28^. The collection of human tissues in compliance with the tissue banking protocol was approved by the Sun Yat-sen University Cancer Center Institutional Review Board, and written informed consent was obtained from each participant. Tumor sections of human and mouse tissues were de-paraffinized and rehydrated, and subjected to antigen retrieval in Target Retrieve Solution (DAKO, S1699) at 95 °C for 20 min. Sections were blocked with 5% horse serum for 1 h at room temperature, incubated with anti-CD31 (1:100, Cell Signaling, 3528, for human tissues), anti-CD31 (1:100, Dianova, DIA-310, for mouse tissues), anti-FSP-1 (1:100, Millipore, 07-2274), anti-NG-2 (1:100, Millipore, AB5320), anti-VEGFR-2 (1:100, Cell Signaling, 9698), or anti-PDGFR-β (1:100, R&D systems, AF1042) antibody overnight at 4 °C. For cell culture staining, the cells were fixed with 4% paraformaldehyde for 15 min and permeabilized with 1% Triton X-100 for 5 min. Cells were blocked with 5% horse serum for 1 h at room temperature, and incubated with anti-NF-κB antibody (Dako, M3527) overnight at 4 °C. and. Sections were stained with Alexa Fluor 488-, 568-, and or 647-conjugated secondary IgGs (1:500, Life Technologies) for 1 h and Alexa Fluor 488-labeled phalloidin (1:100, Invitrogen, A12379) for 20 min at room temperature. Images were acquired with an AxioImager microscope (Zeiss) equipped with AxioCam 506 monochrome charge-coupled devise (CCD) camera (Zeiss). For histological study, tumor tissue sections were stained with hematoxylin and eosin, and imaged with an AxioLab microscope (Zeiss) equipped with AxioCam HRC CCD camera (Zeiss).

### Real-time reverse trascription-PCR analysis

The total RNA was extracted with TRIzol (Invitrogen), isolated with chloroform (Sigma) and 2-propanol (Fisher Scientific), and subjected to reverse transcription with SuperScript III First-Strand Synthesis SuperMix (Life Technologies). Real-time PCR was performed in a 20-μl reaction volume using Fast SYBR^®^ Green Master Mix (Applied Biosystems) and primers: Snail (FP: 5′-AATCGGAAGCCTAACTACAGCGAG-3′, RP: 5′-CCTTGGCCTCAGAGAGCTGG-3′); Slug (FP: 5′-TGTTGCAGTGAGGGCAAGAA-3′, RP: 5′-GACCCTGGTTGCTTCAAGGA-3′); FSP-1 (FP: 5′-TCTCTCCTCAGCGCTTC-3′, RP: 5′-ATAGCAACAGCGTGTGCA-3′); and GAPDH (FP: 5′-GTCTCCTCTGACTTCAACAGCG-3′, RP: 5′-ACCACCCTGTTGCTGTAGCCAA-3′).

### Cell proliferation assay

ECs pretreated with glioma-CM or control medium were trypsinized and seeded on 96-well plates at a density of 2500 cells/well, and allowed to attach for 4 h. Cell viability was determined by Cell-Titer assay (Promega, G7571) according to the manufacturer’s instruction. Luminescence was detected by using a luminescent plate reader (Synergy H4 Hybrid, BioTek).

### siRNA treatment

ECs at 50% confluence were transfected with non-targeting control siRNA (Qiagen, 1027280) or siRNAs targeting Snail (Life Technologies, s13185), Erg-1 (Life Technologies, s194569), NF-κB (Cell Signaling, 6261), PDGFR-α (Life Technologies, s10234), or PDGFR-β (Life Technologies, s10242) using Lipofectamine 2000 (Life Technologies, 11668-019) in serum-free Opti-MEM medium (GIBCO, 31985-070) for 12 h, followed by recovery with serum-supplemented medium for 24 h.

### Immunoblot analysis

Cells were lysed with a NP-40 lysis buffer containing protease inhibitor cocktail (Roche, 11697498001), followed by measurement of total protein concentration. A total 20 μg protein of the lysates was resolved by 4–15% precast SDS-polyacrylamide gel electrophoresis gel (Bio-Rad). After transfer, polyvinylidene fluoride membranes were blotted with anti-GAPDH (Cell Signaling, 5174), anti-FSP-1 (Millipore, 07-2274, Abcam, ab27957, and Abnova H00006275-M01), anti-VEGFR-2 (Cell Signaling, 9698), anti-N-cadherin (Cell Signaling, 13116), anti-α-SMA (Abcam, ab5694), anti-NF-κB (Cell Signaling, 8242), anti-Snail (Cell Signaling, 3879s), anti-Erg (Cell Signaling, 97249), anti-Slug (Cell Signaling, 9585s), anti-PDGFR-α (Cell Signaling, 3164), anti-PDGFR-β (Cell Signaling, 3169), anti-PDGF-AA (Millipore, 07-1436), and anti-PDGF-BB (Millipore, 07-1437) antibodies at 1:1000 dilution. Proteins were detected with horseradish peroxidase-conjugated antibodies specific for either rabbit or mouse IgG (Bio-Rad), followed by ECL development (GE Healthcare, RPN2232). Uncropped scans of all immunoblots are provided in Supplementary Fig. 9.

### ChIP assay

Human brain microvascular ECs were treated with 100 ng/ml PDGF-AB, followed by ChIP assay with a Magna ChIP Kit (Millipore, 17-610). Briefly, the cells were fixed with 1% formaldehyde. The cells were collected by scratching, lyzed, and suspended in nuclear buffer. Five cycles of continuously sonication for 10 × 2 s were applied to break the chromatin into fragments between 100 and 500 bp. The samples were incubated with anti-NF-κB antibody (Cell Signaling, 8242) or anti-Snail antibody (R&D systems, AF3639) and magnetic beads overnight at 4 °C. Normal IgG was used as a negative control. The immunoprecipitants were separated by magnetic rack and washed. The DNA fragments were released by incubation with proteinase K at 62 °C for 2 h with continuously shaking, and isolated by filtration. Real-time PCR was performed in a 20-μl reaction volume using Fast SYBR Green Master Mix (Applied Biosystems) and primers of Snail or VEGFR-2 promoter including Snail FP #1: −633 to −614, 5′-TGCTGGGCGCTCCGTAAACA; Snail RP #1: −526 to −545, 5′-TGGCTCTCGGCGGCTTGAAA; Snail FP #2: −1184 to −1165, 5′-CCCCTATGGAGCCGTGTTAC; and Snail RP #2: −1023 to −1042, 5′-GCCACCGAAGGATTTTCAGC. For electrophoresis analysis, PCR was performed with a NEB Next High-Fidelity 2X PCR Master Mix (New England Biolabs) and Snail primers #2 or VEGFR-2 primers (FP: −1500 to −1481, 5′-AGAGGAGGAAGGCCACTCTT; RP: −1311 to −1330, 5′-GTCAGCTTAGGTGCCTCCAT), and DNA was resolved by agarose electrophoresis, followed by gel imaging.

### Statistics

Student’s *t* test (unpaired, two-tailed) and log-rank (Mantel–Cox test) analysis were performed by using Prism software for statistical analysis between groups, and *P* < 0.05 was considered to represent a statistically significant difference.

### Data availability

RNA-seq data have been deposited in NCBI’s Gene Expression Omnibus under the accession GSE115850. All data are available within the Article and Supplementary Files, or available from the authors upon request.

## Electronic supplementary material

Supplementary Information

## References

[1] Carmeliet P, Jain RK (2011). Principles and mechanisms of vessel normalization for cancer and other angiogenic diseases. Nat. Rev. Drug Discov..

[2] Weis SM, Cheresh DA (2011). Tumor angiogenesis: molecular pathways and therapeutic targets. Nat. Med..

[3] Hanahan D, Weinberg RA (2011). Hallmarks of cancer: the next generation. Cell.

[4] Beck B (2011). A vascular niche and a VEGF-Nrp1 loop regulate the initiation and stemness of skin tumours. Nature.

[5] Butler JM, Kobayashi H, Rafii S (2010). Instructive role of the vascular niche in promoting tumour growth and tissue repair by angiocrine factors. Nat. Rev. Cancer.

[6] Calabrese C (2007). A perivascular niche for brain tumor stem cells. Cancer Cell.

[7] Carmeliet P, Jain RK (2011). Molecular mechanisms and clinical applications of angiogenesis. Nature.

[8] Charles N (2010). Perivascular nitric oxide activates notch signaling and promotes stem-like character in PDGF-induced glioma cells. Cell Stem Cell.

[9] Ghajar CM (2013). The perivascular niche regulates breast tumour dormancy. Nat. Cell Biol..

[10] Lu J (2013). Endothelial cells promote the colorectal cancer stem cell phenotype through a soluble form of jagged-1. Cancer Cell.

[11] Cao Z (2014). Angiocrine factors deployed by tumor vascular niche induce B cell lymphoma invasiveness and chemoresistance. Cancer Cell.

[12] Folkman J (1971). Tumor angiogenesis: therapeutic implications. N. Engl. J. Med..

[13] Bergers G, Hanahan D (2008). Modes of resistance to anti-angiogenic therapy. Nat. Rev. Cancer.

[14] Huang M (2016). c-Met-mediated endothelial plasticity drives aberrant vascularization and chemoresistance in glioblastoma. J. Clin. Invest..

[15] Stupp R (2005). Radiotherapy plus concomitant and adjuvant temozolomide for glioblastoma. N. Engl. J. Med..

[16] Huse JT, Holland EC (2010). Targeting brain cancer: advances in the molecular pathology of malignant glioma and medulloblastoma. Nat. Rev. Cancer.

[17] Gilbert MR (2014). A randomized trial of bevacizumab for newly diagnosed glioblastoma. N. Engl. J. Med..

[18] Batchelor TT (2007). AZD2171, a pan-VEGF receptor tyrosine kinase inhibitor, normalizes tumor vasculature and alleviates edema in glioblastoma patients. Cancer Cell.

[19] Kim KJ (1993). Inhibition of vascular endothelial growth factor-induced angiogenesis suppresses tumour growth in vivo. Nature.

[20] Friedman HS (2009). Bevacizumab alone and in combination with irinotecan in recurrent glioblastoma. J. Clin. Oncol..

[21] Chinot OL (2014). Bevacizumab plus radiotherapy-temozolomide for newly diagnosed glioblastoma. N. Engl. J. Med..

[22] Ferrara N, Gerber HP, LeCouter J (2003). The biology of VEGF and its receptors. Nat. Med..

[23] Olsson AK, Dimberg A, Kreuger J, Claesson-Welsh L (2006). VEGF receptor signalling—in control of vascular function. Nat. Rev. Mol. Cell Biol..

[24] Simons M, Gordon E, Claesson-Welsh L (2016). Mechanisms and regulation of endothelial VEGF receptor signalling. Nat. Rev. Mol. Cell Biol..

[25] Andrae J, Gallini R, Betsholtz C (2008). Role of platelet-derived growth factors in physiology and medicine. Genes Dev..

[26] Donovan J, Abraham D, Norman J (2013). Platelet-derived growth factor signaling in mesenchymal cells. Front Biosci. (Landmark Ed.).

[27] Fan Y (2014). Profilin-1 phosphorylation directs angiocrine expression and glioblastoma progression through HIF-1alpha accumulation. Nat. Cell Biol..

[28] Wang Q (2018). Vascular niche IL-6 induces alternative macrophage activation in glioblastoma through HIF-2alpha. Nat. Commun..

[29] Osterreicher CH (2011). Fibroblast-specific protein 1 identifies an inflammatory subpopulation of macrophages in the liver. Proc. Natl Acad. Sci. USA.

[30] De Palma M (2005). Tie2 identifies a hematopoietic lineage of proangiogenic monocytes required for tumor vessel formation and a mesenchymal population of pericyte progenitors. Cancer Cell.

[31] Teichert M (2017). Pericyte-expressed Tie2 controls angiogenesis and vessel maturation. Nat. Commun..

[32] Giannini G (2003). EGF- and cell-cycle-regulated STAG1/PMEPA1/ERG1.2 belongs to a conserved gene family and is overexpressed and amplified in breast and ovarian cancer. Mol. Carcinog..

[33] Romashkova JA, Makarov SS (1999). NF-kappaB is a target of AKT in anti-apoptotic PDGF signalling. Nature.

[34] Zeisberg EM, Potenta S, Xie L, Zeisberg M, Kalluri R (2007). Discovery of endothelial to mesenchymal transition as a source for carcinoma-associated fibroblasts. Cancer Res..

[35] Scully S (2012). Transdifferentiation of glioblastoma stem-like cells into mural cells drives vasculogenic mimicry in glioblastomas. J. Neurosci..

[36] Ricci-Vitiani L (2010). Tumour vascularization via endothelial differentiation of glioblastoma stem-like cells. Nature.

[37] Wang R (2010). Glioblastoma stem-like cells give rise to tumour endothelium. Nature.

[38] Soda Y (2011). Transdifferentiation of glioblastoma cells into vascular endothelial cells. Proc. Natl Acad. Sci. USA.

[39] Wu HB (2017). Autophagy-induced KDR/VEGFR-2 activation promotes the formation of vasculogenic mimicry by glioma stem cells. Autophagy.

[40] Heldin CH, Westermark B (1999). Mechanism of action and in vivo role of platelet-derived growth factor. Physiol. Rev..

[41] Bergers G, Song S, Meyer-Morse N, Bergsland E, Hanahan D (2003). Benefits of targeting both pericytes and endothelial cells in the tumor vasculature with kinase inhibitors. J. Clin. Invest..

[42] Jaffe GJ (2017). Dual antagonism of PDGF and VEGF in neovascular age-related macular degeneration: a phase IIb, multicenter, randomized controlled trial. Ophthalmology.

[43] Jain RK (2005). Normalization of tumor vasculature: an emerging concept in antiangiogenic therapy. Science.

[44] Jain RK, Lahdenranta J, Fukumura D (2008). Targeting PDGF signaling in carcinoma-associated fibroblasts controls cervical cancer in mouse model. PLoS Med..

[45] Potenta S, Zeisberg E, Kalluri R (2008). The role of endothelial-to-mesenchymal transition in cancer progression. Br. J. Cancer.

[46] Maddaluno L (2013). EndMT contributes to the onset and progression of cerebral cavernous malformations. Nature.

[47] Kalluri R, Weinberg RA (2009). The basics of epithelial-mesenchymal transition. J. Clin. Invest..

[48] Thiery JP (2002). Epithelial-mesenchymal transitions in tumour progression. Nat. Rev. Cancer.

[49] Lamouille S, Xu J, Derynck R (2014). Molecular mechanisms of epithelial-mesenchymal transition. Nat. Rev. Mol. Cell Biol..

[50] Bachelder RE, Yoon SO, Franci C, de Herreros AG, Mercurio AM (2005). Glycogen synthase kinase-3 is an endogenous inhibitor of Snail transcription: implications for the epithelial-mesenchymal transition. J. Cell Biol..

[51] Pires BR (2017). NF-kappaB is involved in the regulation of EMT genes in breast cancer cells. PLoS ONE.

[52] Xu X (2015). Snail is a direct target of hypoxia-inducible factor 1alpha (HIF1alpha) in hypoxia-induced endothelial to mesenchymal transition of human coronary endothelial cells. J. Biol. Chem..

[53] Kokudo T (2008). Snail is required for TGFbeta-induced endothelial-mesenchymal transition of embryonic stem cell-derived endothelial cells. J. Cell Sci..

[54] Medici D, Potenta S, Kalluri R (2011). Transforming growth factor-beta2 promotes Snail-mediated endothelial-mesenchymal transition through convergence of Smad-dependent and Smad-independent signalling. Biochem. J..

[55] Mahmoud MM (2017). Shear stress induces endothelial-to-mesenchymal transition via the transcription factor Snail. Sci. Rep..

[56] Peinado H, Olmeda D, Cano A (2007). Snail, Zeb and bHLH factors in tumour progression: an alliance against the epithelial phenotype?. Nat. Rev. Cancer.

[57] Nieto MA (2002). The snail superfamily of zinc-finger transcription factors. Nat. Rev. Mol. Cell Biol..

[58] Cheng JC, Chang HM, Leung PC (2013). Transforming growth factor-beta1 inhibits trophoblast cell invasion by inducing Snail-mediated down-regulation of vascular endothelial-cadherin protein. J. Biol. Chem..

[59] Lopez D, Niu G, Huber P, Carter WB (2009). Tumor-induced upregulation of Twist, Snail, and Slug represses the activity of the human VE-cadherin promoter. Arch. Biochem. Biophys..

[60] Wang Y (2010). Ephrin-B2 controls VEGF-induced angiogenesis and lymphangiogenesis. Nature.

[61] Liu Y (2007). Somatic cell type specific gene transfer reveals a tumor-promoting function for p21(Waf1/Cip1). EMBO J..

[62] Ciznadija D, Liu Y, Pyonteck SM, Holland EC, Koff A (2011). Cyclin D1 and cdk4 mediate development of neurologically destructive oligodendroglioma. Cancer Res..

