# Supplementary material for: PDGF-mediated mesenchymal transformation renders endothelial resistance to anti-VEGF treatment in glioblastoma
Source: Nat Commun. 2018 Aug 27;9:3439. doi: 10.1038/s41467-018-05982-z (PMC6110798; doi:10.1038/s41467-018-05982-z)
Supplement: Supplementary file 1 — Supplementary Information [file 41467_2018_5982_MOESM1_ESM.pdf]

Supplementary Information

PDGF-mediated mesenchymal transformation renders endothelial resistance to anti-VEGF treatment in glioblastoma

Liu et al.

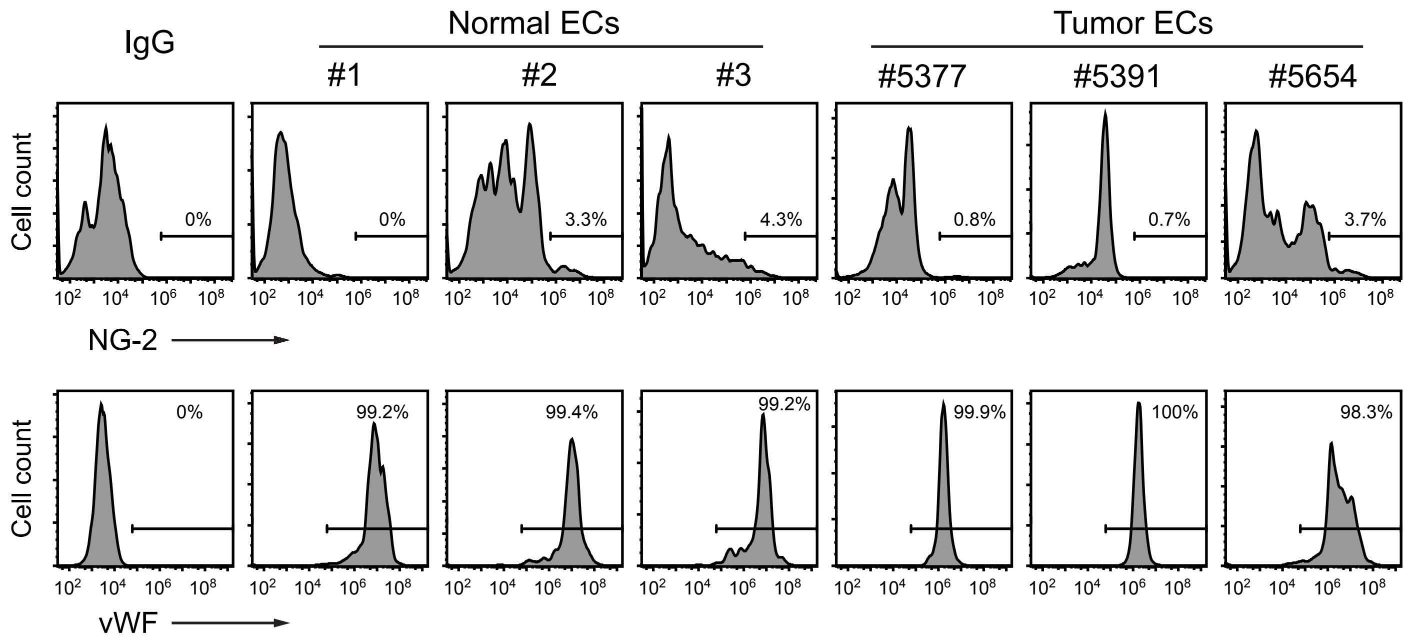

Supplementary Figure 1. Identification of microvascular ECs isolated from normal human brains and GBM tumors. ECs derived from normal human brains (#1 and #2 from adult brains and #3 from fetal brain) and from human GBM tumors (patients #5377, #5391,and #5654) were immunostained with anti-NG-2 or anti-vWF antibody or isotype IgG, followed by flow cytometry analysis. Representative sortings are shown.

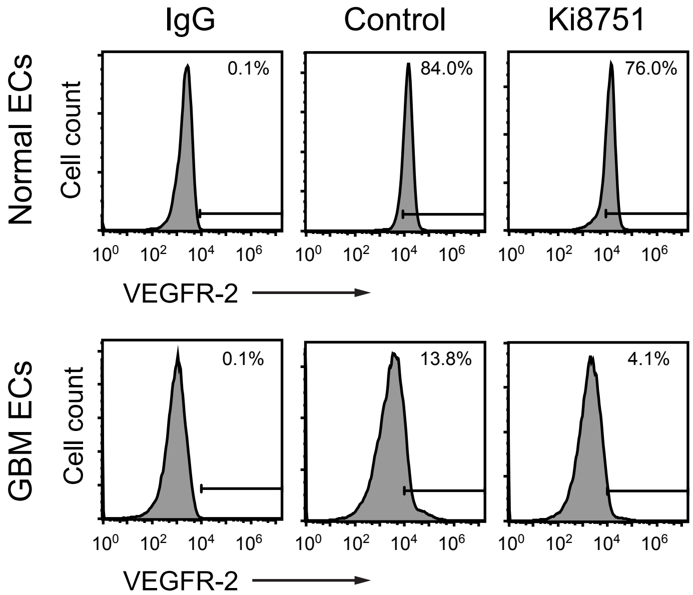

Supplementary Figure 2. VEGFR-2 expression after Ki8751 treatment in ECs.
ECs derived from normal human brain and from human GBM tumor (patients #5377) were treated with 1 μM Ki8751 or control medium. Single cell suspensions were immunostained with anti-VEGFR-2 antibody or isotype IgG, followed by flow cytometry analysis. Representative sortings are shown.

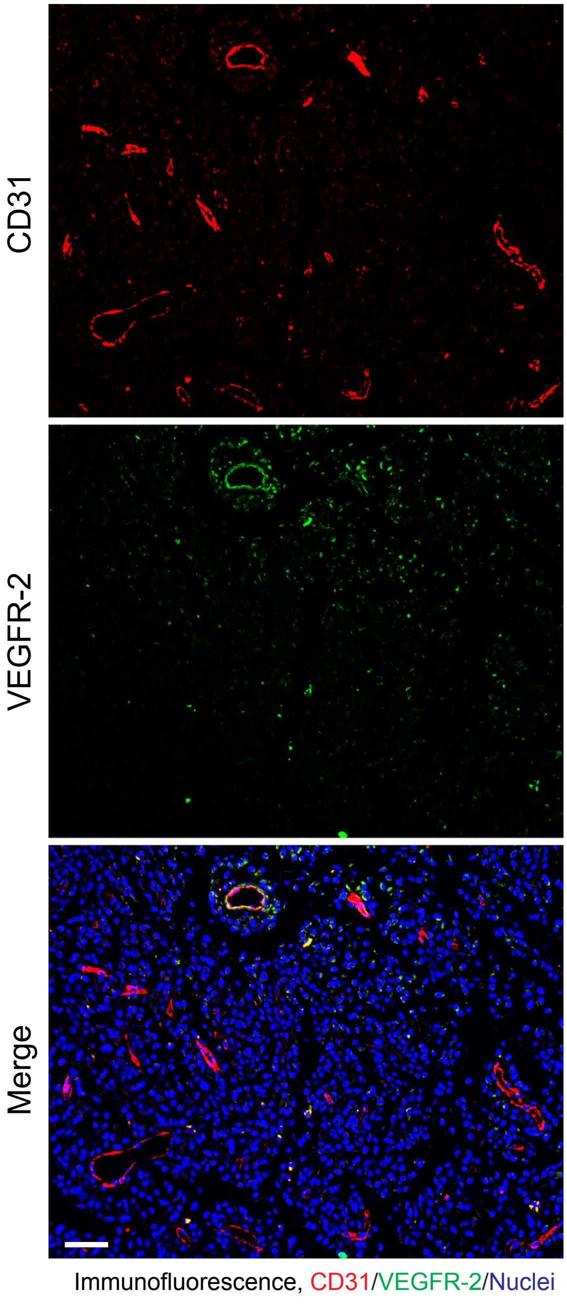

Supplementary Figure 3. Diminished expression of VEGFR-2 in GBM-associated ECs.
Tissue sections from surgical specimens of human patients with GBM (n = 3) were stained with anti-CD31 and anti-VEGFR-2 antibodies, followed by immunofluorescence analysis. Representative images are shown. Scale bar: 100 μm.

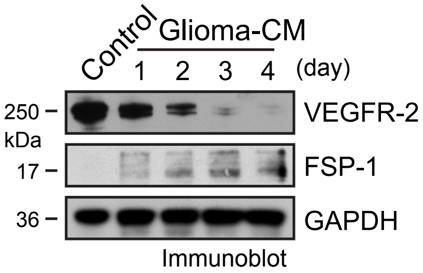

Supplementary Figure 4. Effects of glioma-CM on Endo-MT in ECs.
Glioma-CM was harvested from the medium of U251 human glioma cells cultured under normoxia. Human brain ECs were treated with glioma-CM or control normal medium. EC lysates were immunoblotted.

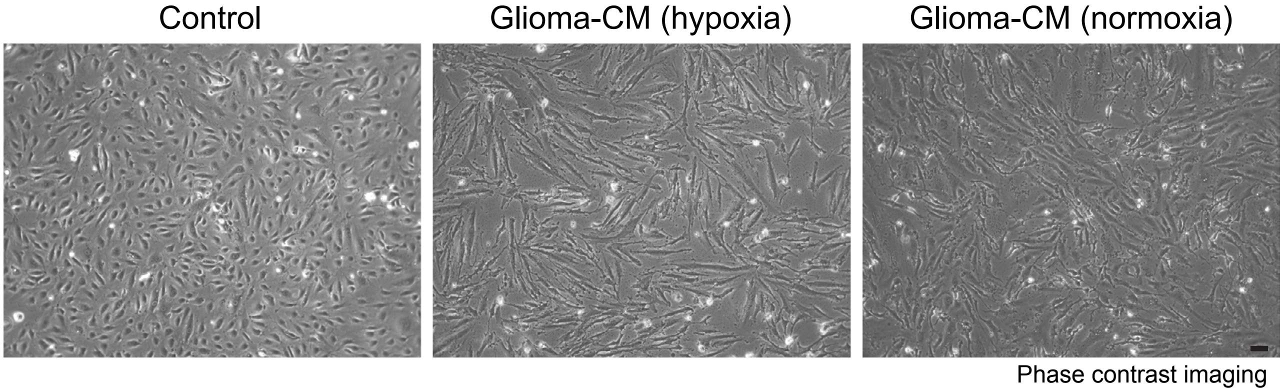

Supplementary Figure 5. Effects of glioma-CM on EC morphology.
Human brain ECs were treated with control normal medium or glioma-CM that was isolated from U251 human glioma cells cultured under hypoxia or normoxia. After 48 h incubation, cells were imaged. Scale bar: 100 μm.

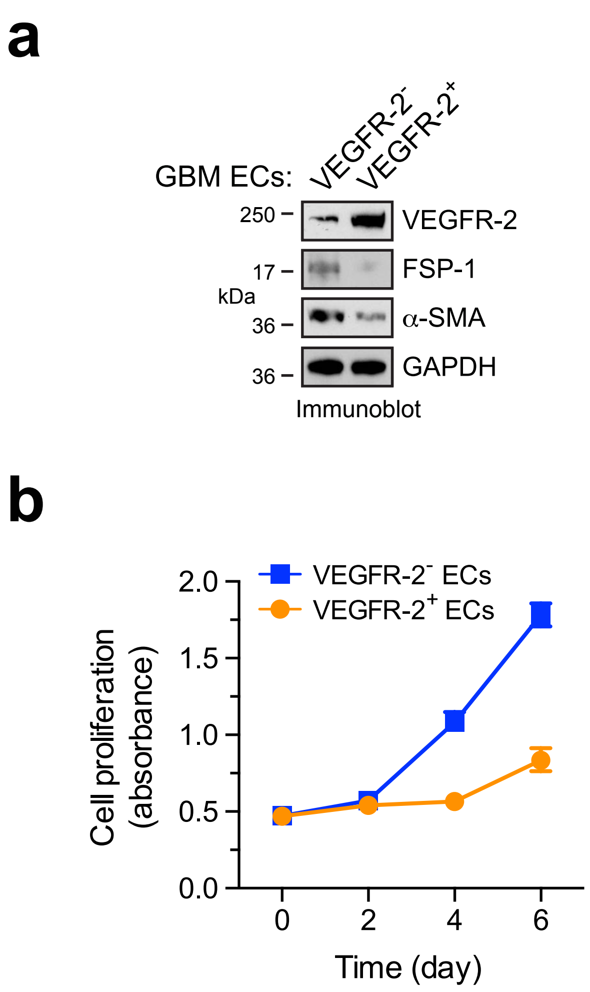

Supplementary Figure 6. VEGFR2$^+$ and VEGFR2$^-$ GBM ECs.
ECs were isolated from the GBM tumor of human patient #5377. Cells were incubated with anti-VEGFR-2 antibody-conjugated magnetic beads, followed by magnetic-activating cell sorting (MACS). VEGFR-2$^+$ and VEGFR-2$^-$ cells were isolated and cultured. (**a**) Cell lysates were analyzed by immunoblot. (**b**) Cells were subjected to proliferation analysis (n = 3, mean ± SD).

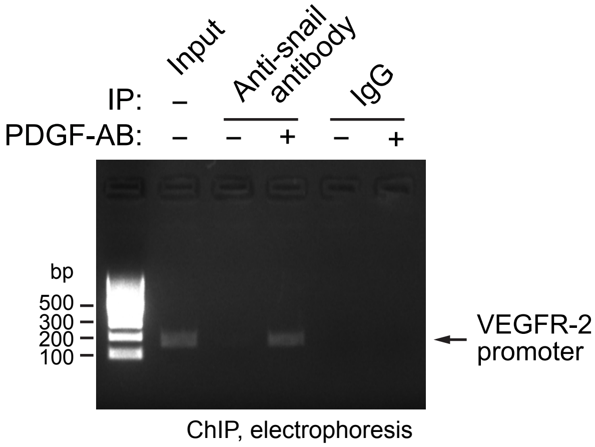

Supplementary Figure 7. PDGF-AB induces Snail binding to VEGFR-2 promoter in ECs.
Human brain ECs were treated with PDGF-AB or control medium for 24 h. Nuclear extracts were immunoprecipitated with anti-Snail antibody or IgG, and subjected to ChIP analysis. DNA was resolved by agarose electrophoresis, and imaged. The arrow indicates the amplified DNA in VEGFR-2 promoter.

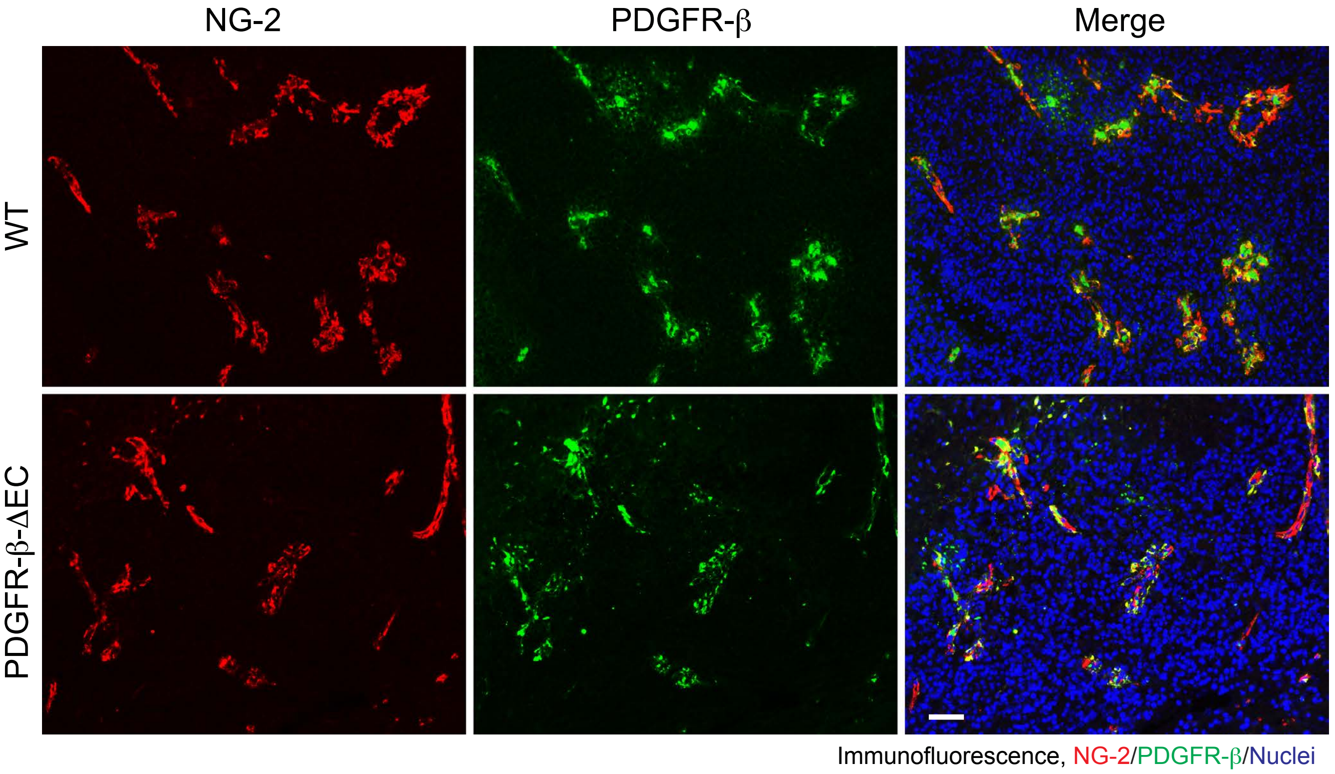

Supplementary Figure 8. Pericyte and PDGFR-β expression in mouse GBM tumors.
The primary GBM was induced in *Ntv-a*;*Ink4a-Arf*$^{-/-}$;*Pten*$^{-/-}$;*LSL-Luc* donor mice by RCAS-mediated somatic gene transfer. Single-cell tumor suspension was implanted into *Pdgfrb*$^{fl/fl}$ (WT) or *Tie2-Cre*;*Pdgfrb*$^{fl/fl}$ (PDGFR-β-ΔEC) recipient mice. Tumor sections were stained with anti-NG-2 and anti-PDGFR-β antibodies, followed by immunofluorescence analysis. Representative images are shown (n = 4 mice). Scale bar: 100 μm.

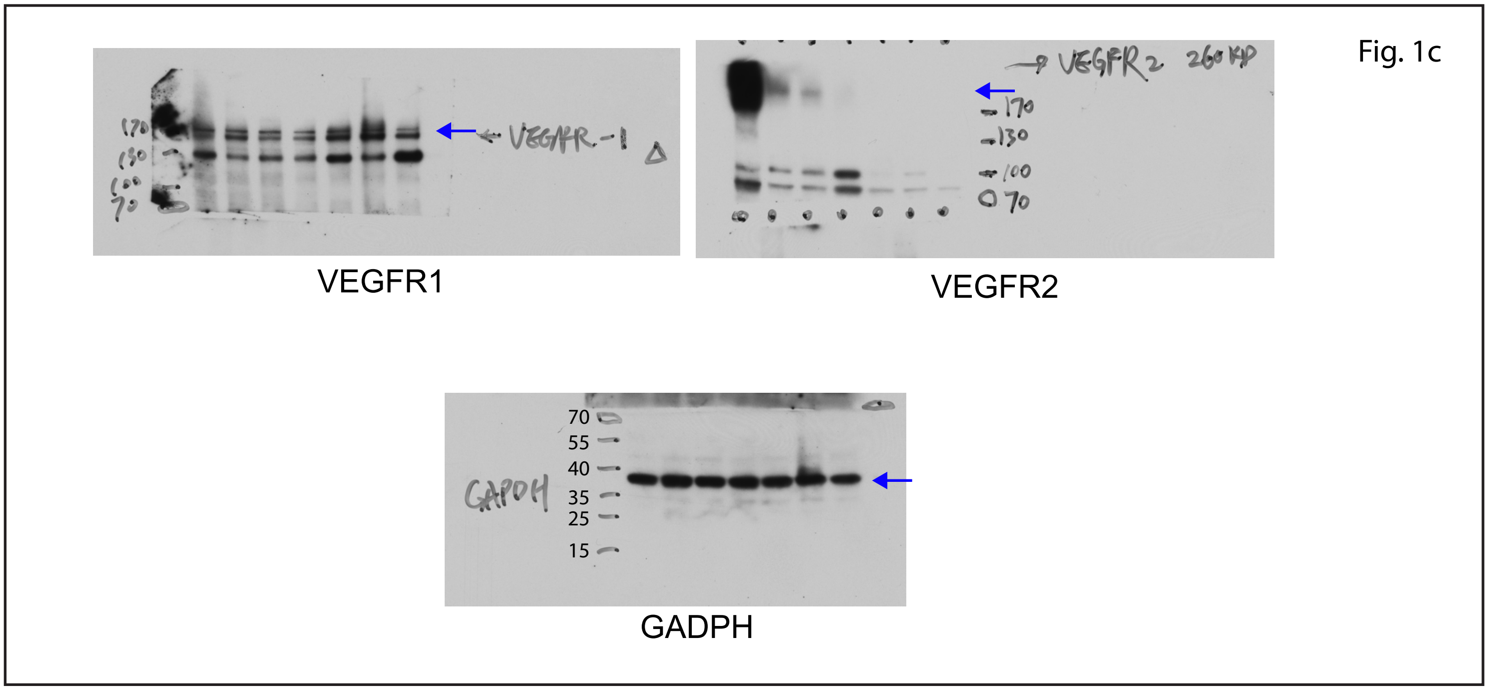

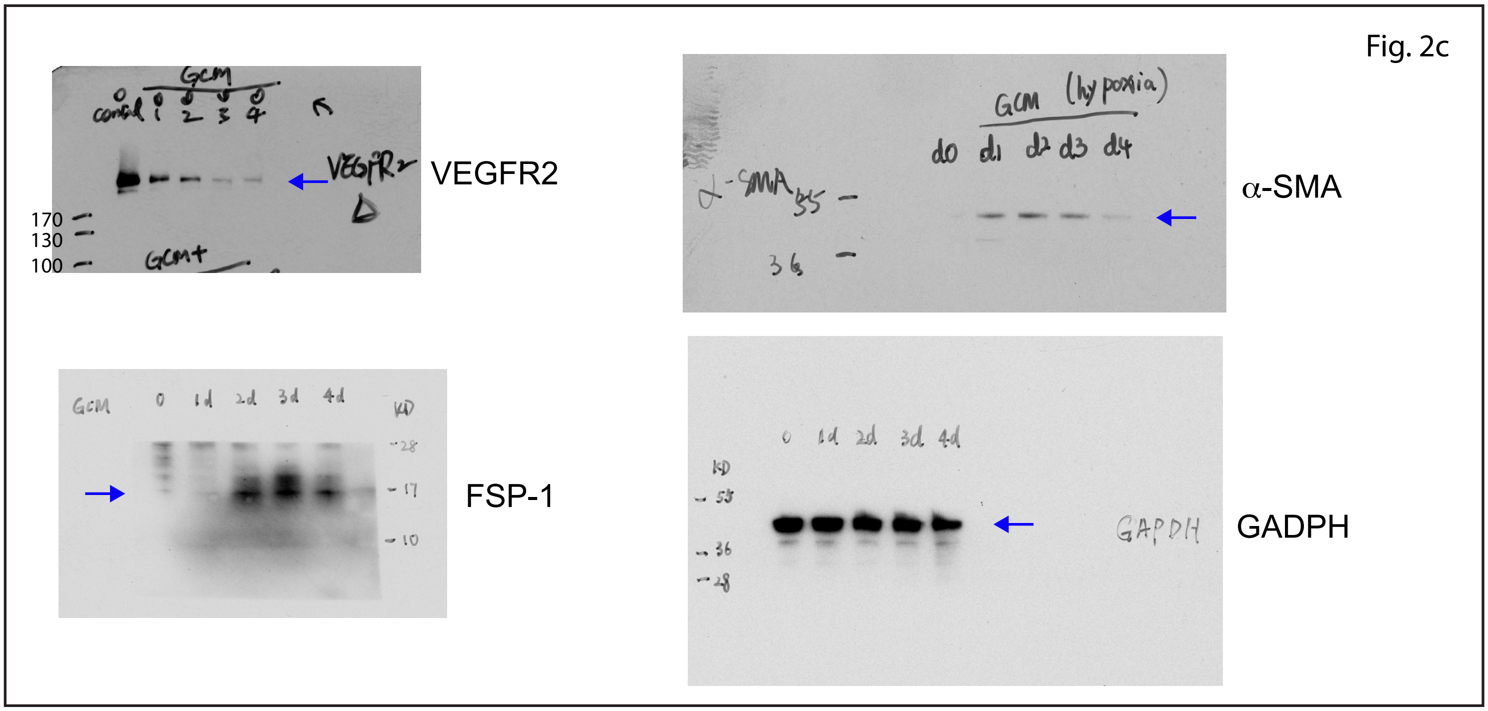

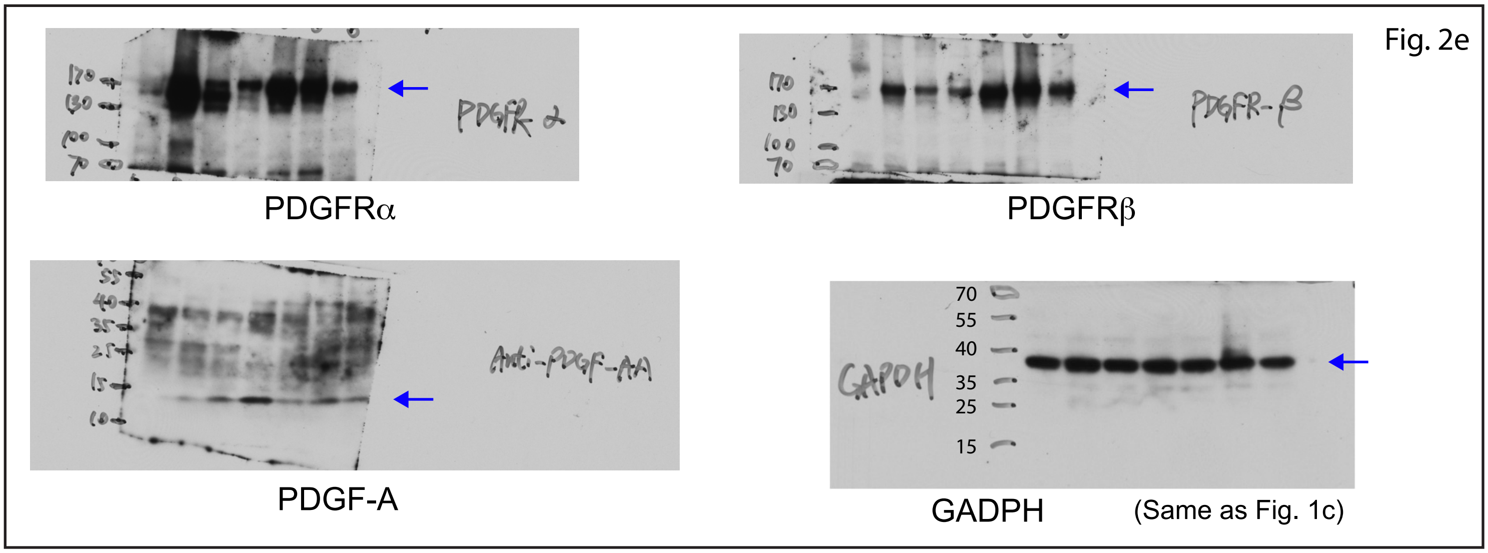

Supplementary Figure 9. Uncropped blots.

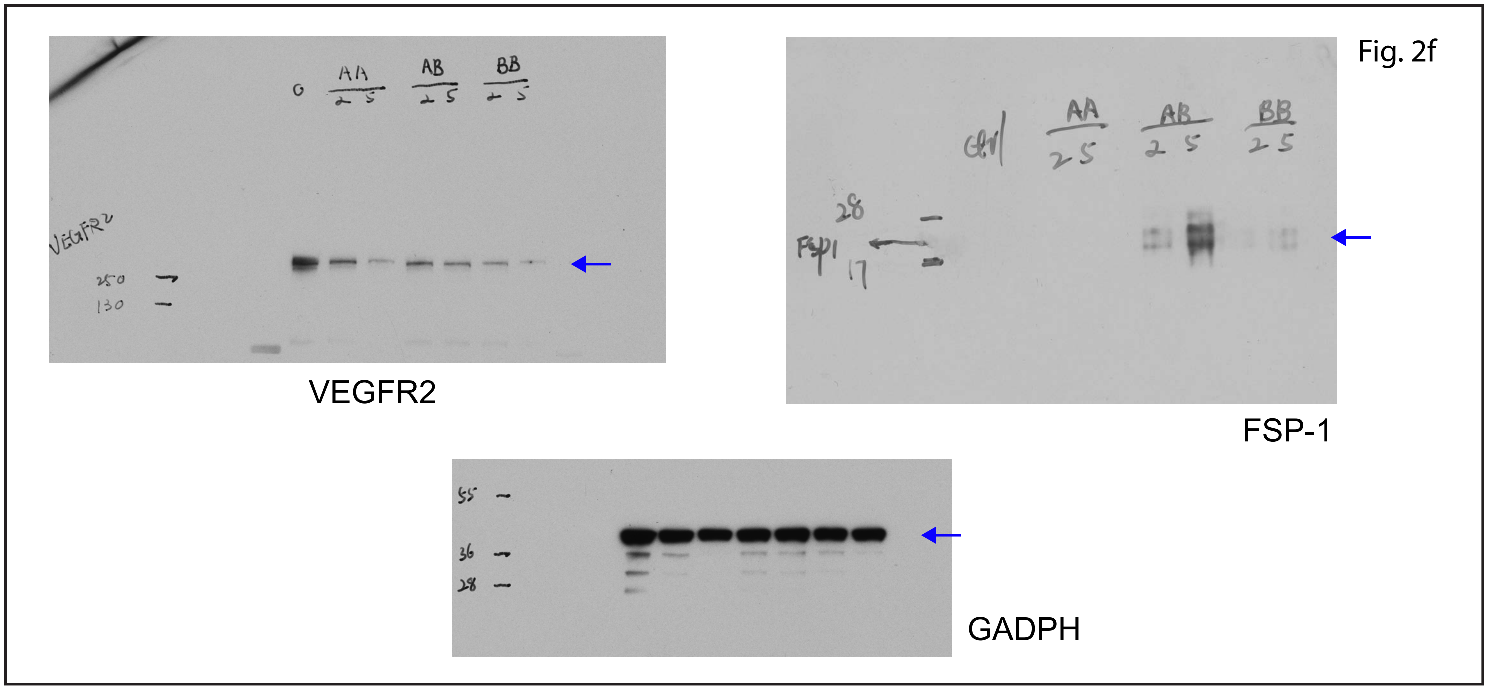

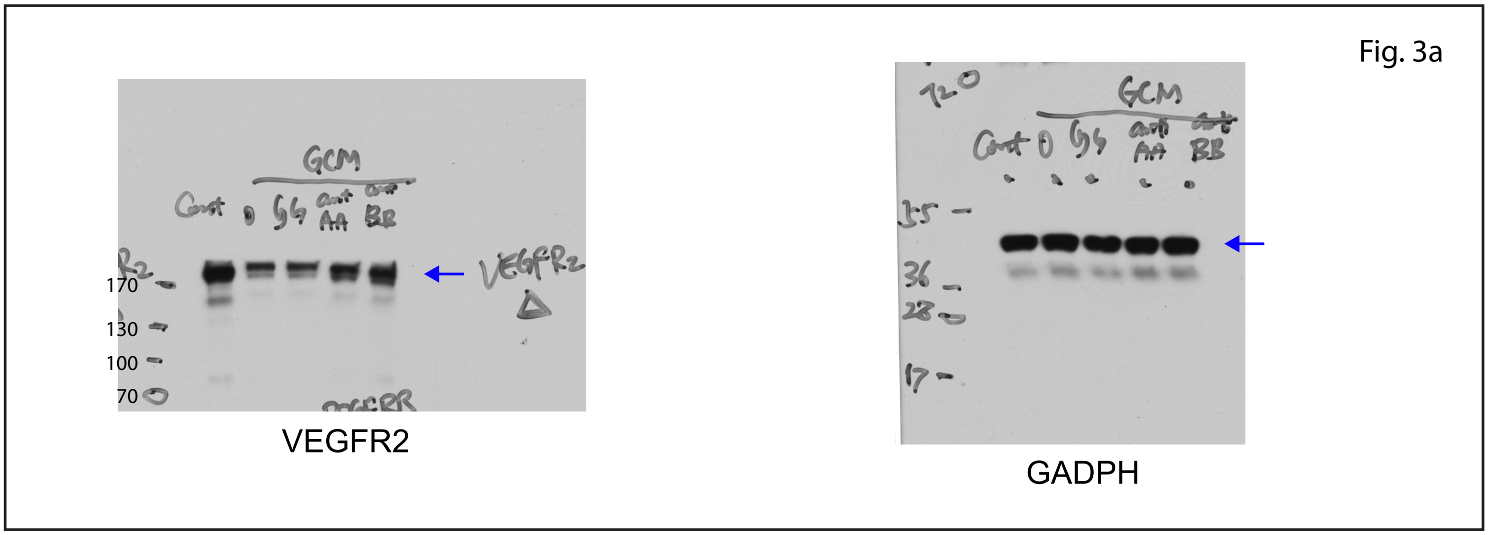

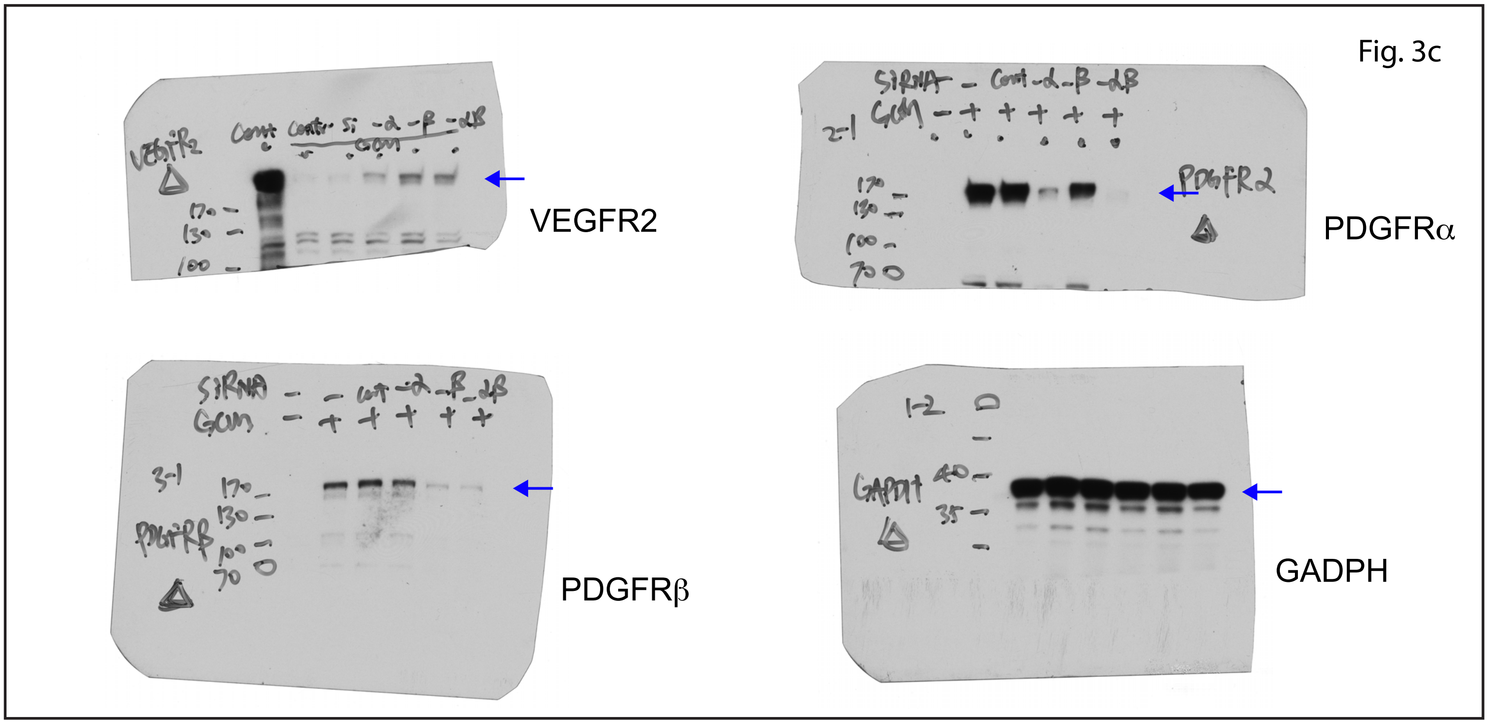

Supplementary Figure 9. Uncropped blots.

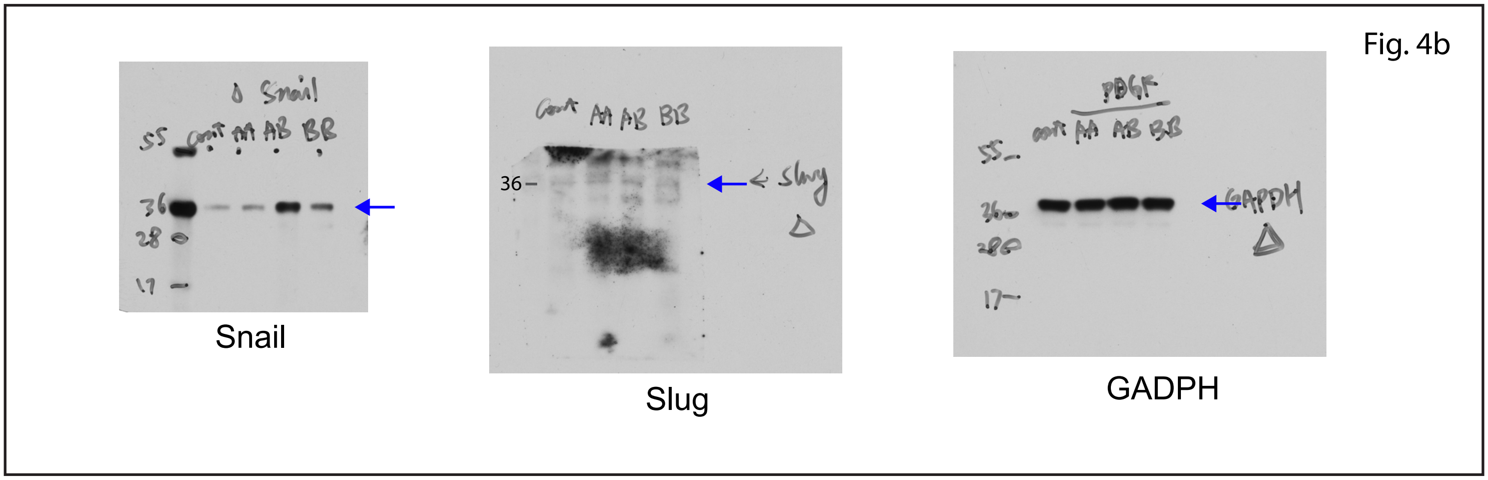

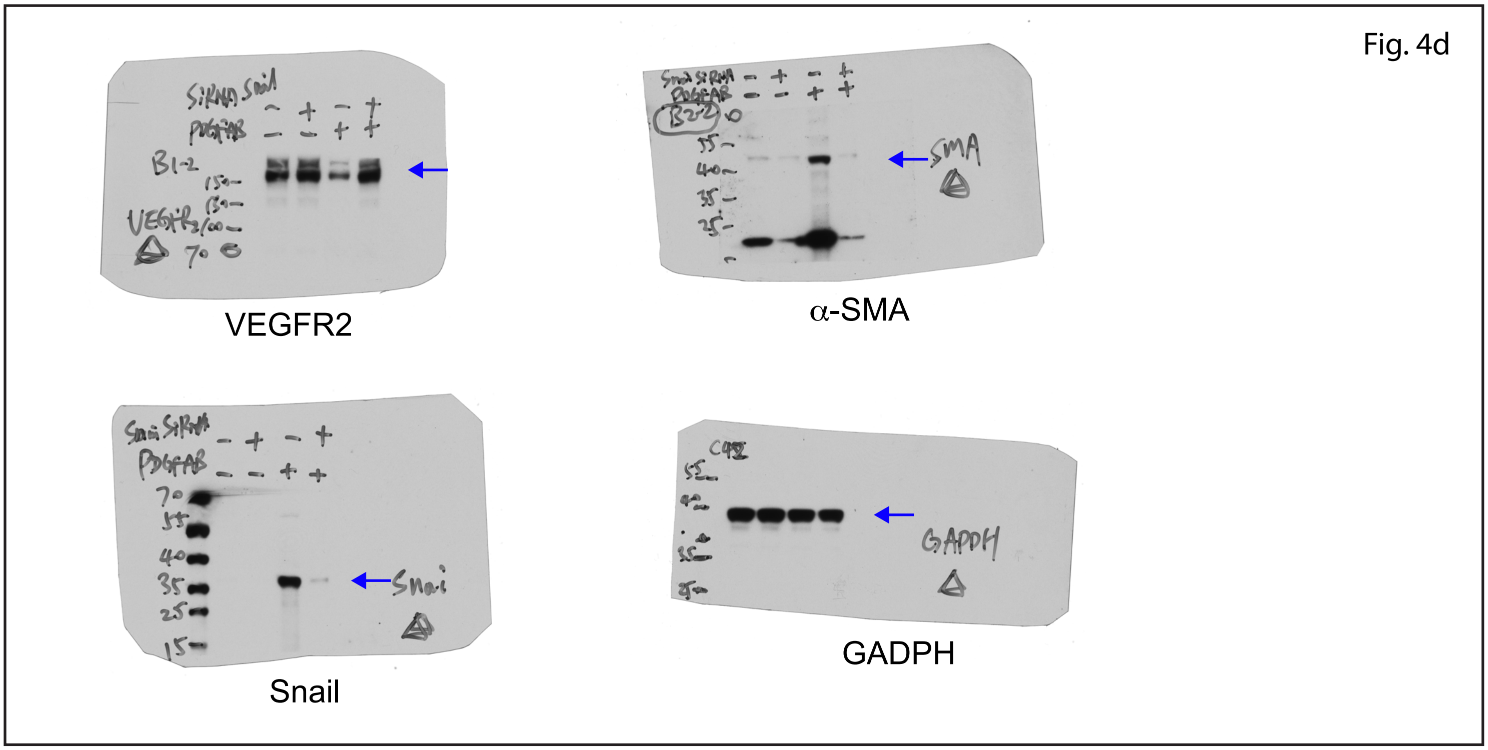

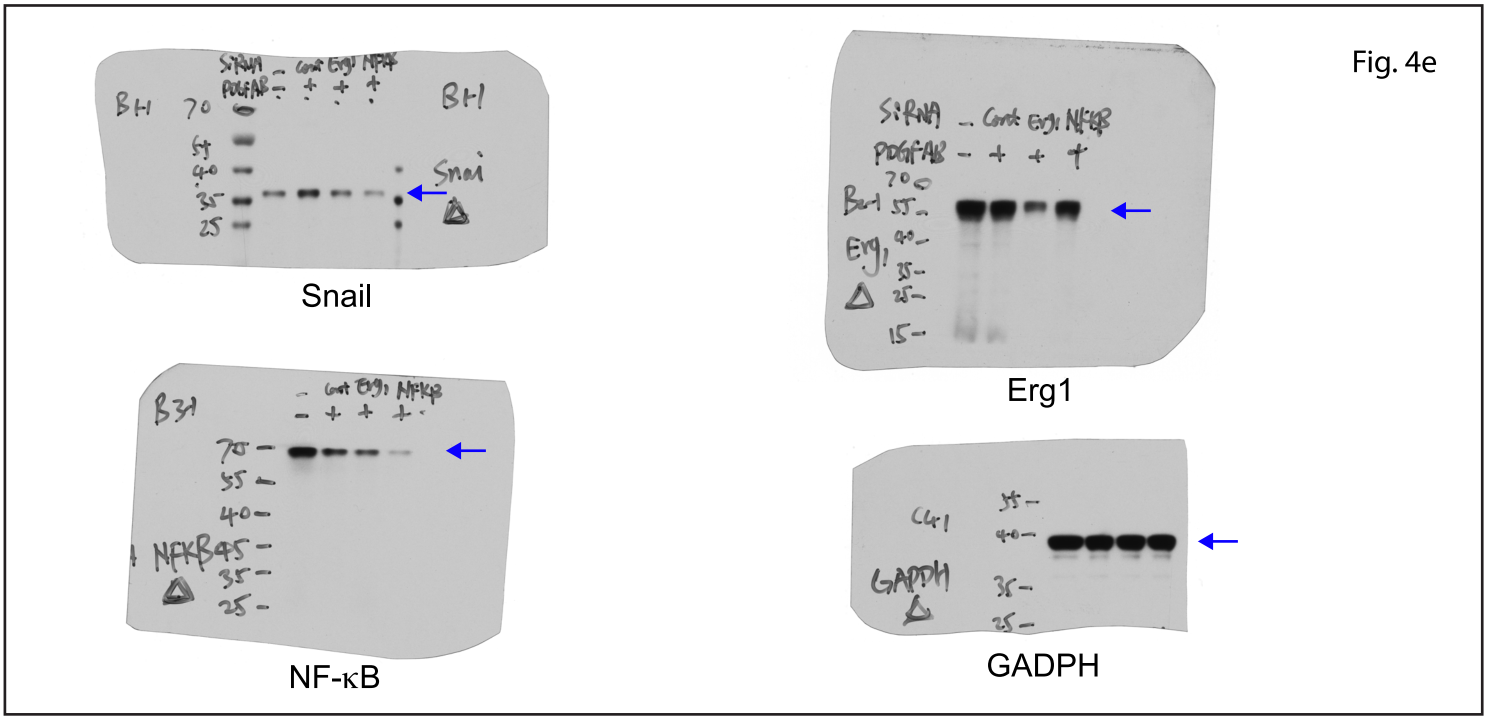

Supplementary Figure 9. Uncropped blots.

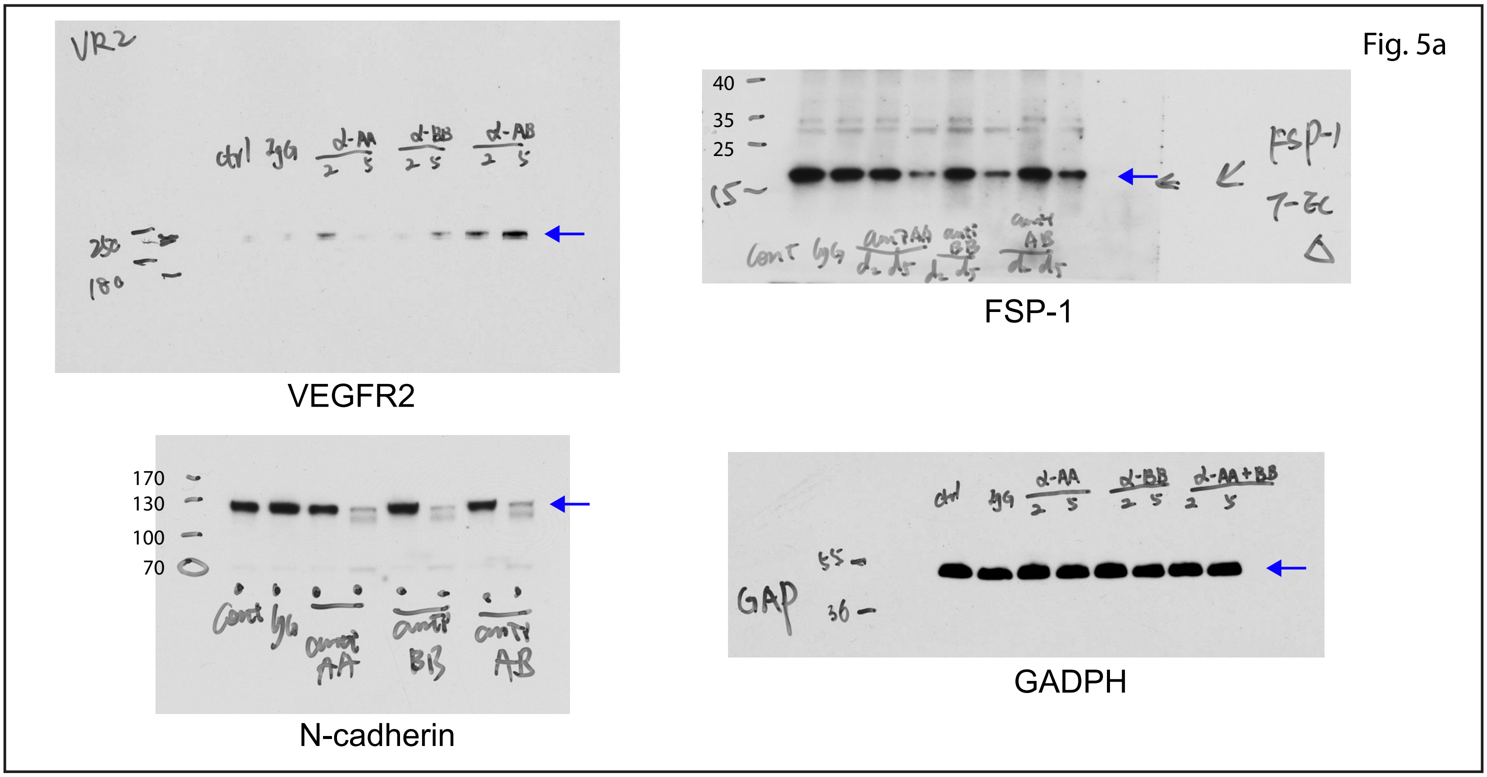

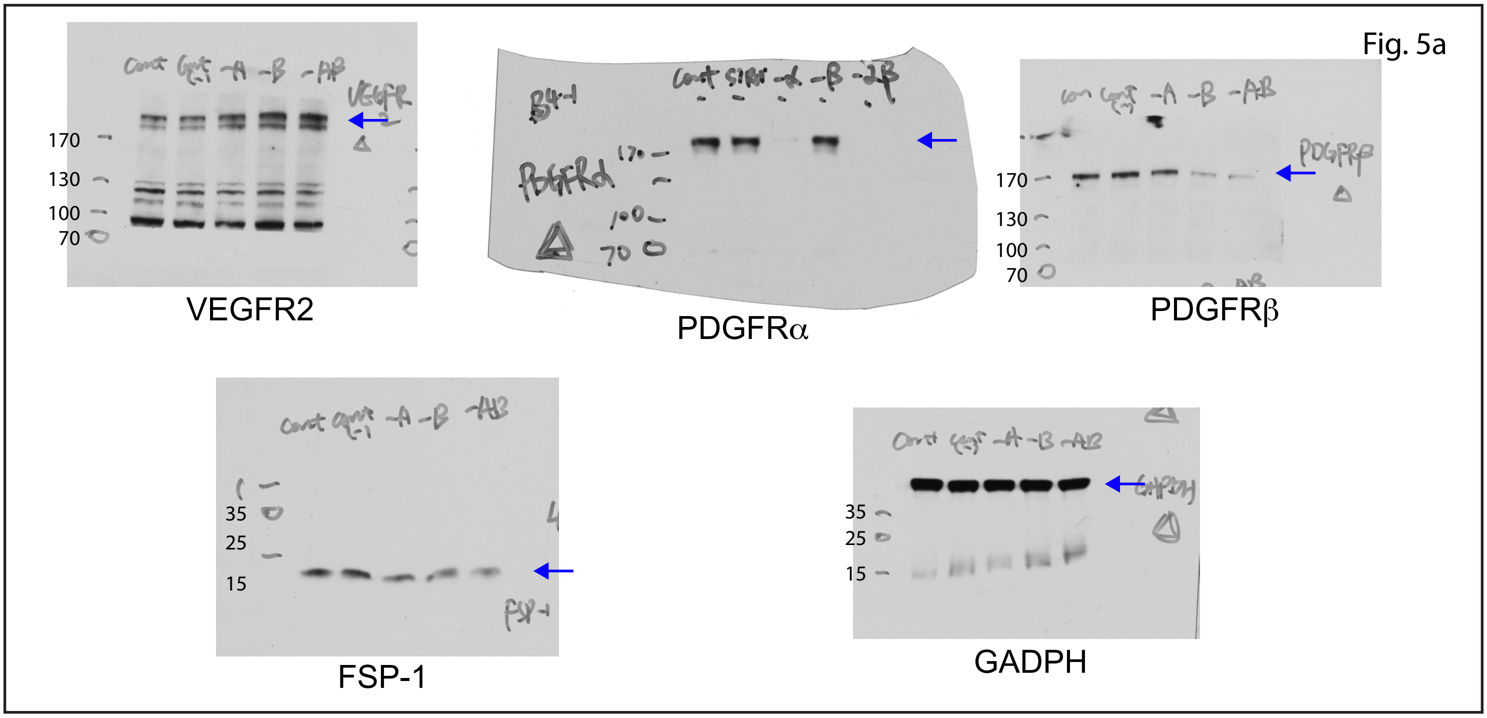

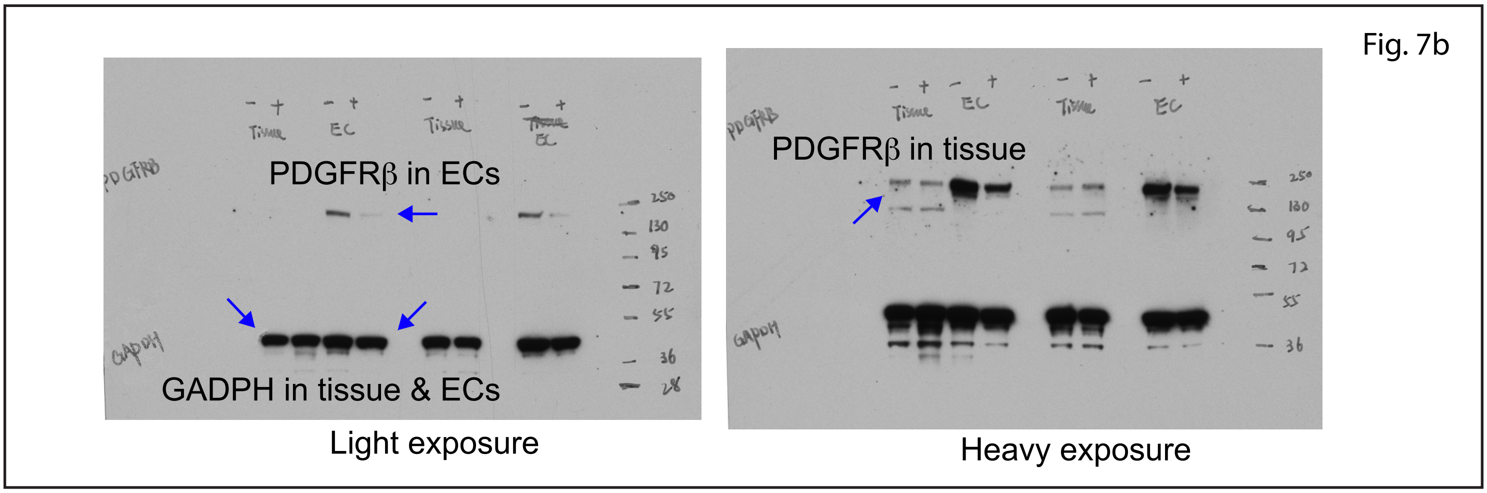

Supplementary Figure 9. Uncropped blots.

Fig. S4

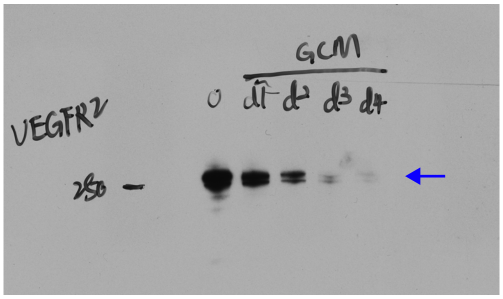

VEGFR2

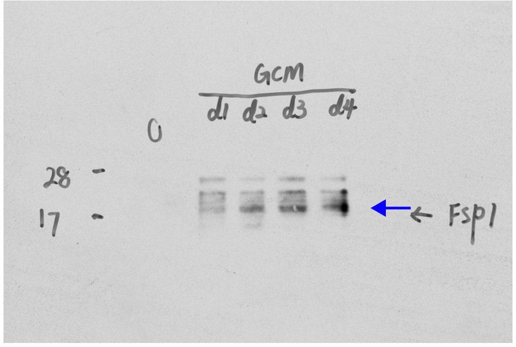

FSP-1

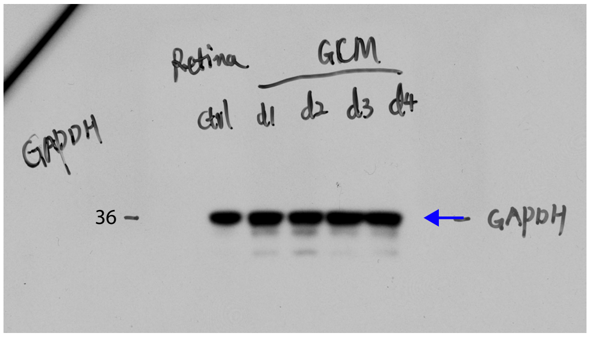

GADPH

Fig. S6

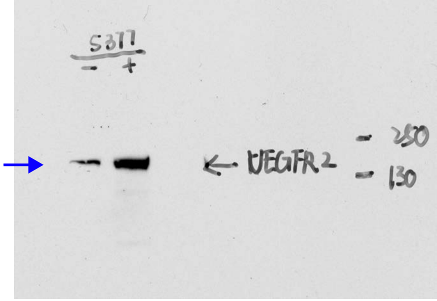

VEGFR2

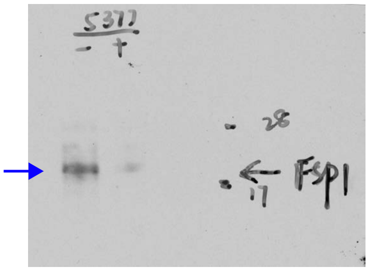

FSP-1

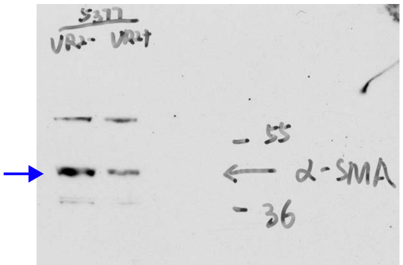

α-SMA

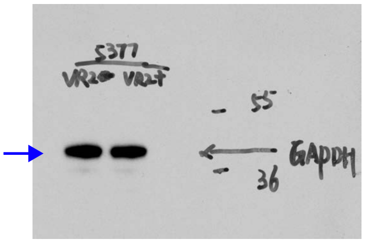

GADPH

Supplementary Figure 9. Uncropped blots.
